# Climate change, land cover change, and overharvesting threaten a widely used medicinal plant in South Africa

**DOI:** 10.1002/eap.2545

**Published:** 2022-03-21

**Authors:** Vivienne P. Groner, Owen Nicholas, Tafadzwanashe Mabhaudhi, Rob Slotow, H. Reşit Akçakaya, Georgina M. Mace, Richard G. Pearson

**Affiliations:** ^1^ Department of Genetics, Evolution and Environment, Centre for Biodiversity and Environment Research University College London London UK; ^2^ Department of Statistical Science University College London London UK; ^3^ Centre for Transformative Agricultural and Food Systems, School of Agricultural, Earth and Environmental Sciences University of Kwazulu‐Natal Pietermaritzburg South Africa; ^4^ International Water Management Institute (IWMI‐GH) Accra Ghana; ^5^ Centre for Transformative Agricultural and Food Systems, School of Life Sciences University of Kwazulu‐Natal Pietermaritzburg South Africa; ^6^ Department of Genetics, Evolution and Environment University College London UK; ^7^ Department of Ecology and Evolution Stony Brook University Stony Brook New York USA; ^8^ IUCN Species Survival Commission IUCN Gland Switzerland

**Keywords:** climate change, *Clivia miniata*, conservation, ecosystem services, land cover change, medicinal plants, metapopulation model, overharvesting, South Africa

## Abstract

Medicinal plants contribute substantially to the well‐being of people in large parts of the world, providing traditional medicine and supporting livelihoods from trading plant parts, which is especially significant for women in low‐income communities. However, the availability of wild medicinal plants is increasingly threatened; for example, the Natal Lily (*Clivia miniata*), which is one of the most widely traded plants in informal medicine markets in South Africa, lost over 40% of individuals over the last 90 years. Understanding the species’ response to individual and multiple pressures is essential for prioritizing and planning conservation actions. To gain this understanding, we simulated the future range and abundance of *C. miniata* by coupling Species Distribution Models with a metapopulation model (RAMAS‐GIS). We contrasted scenarios of climate change (RCP2.6 vs. RCP8.5), land cover change (intensification vs. expansion), and harvesting (only juveniles vs. all life‐stages). All our scenarios pointed to continuing declines in suitable habitat and abundance by the 2050s. When acting independently, climate change, land cover change, and harvesting each reduced the projected abundance substantially, with land cover change causing the most pronounced declines. Harvesting individuals from all life stages affected the projected metapopulation size more negatively than extracting only juveniles. When the three pressures acted together, declines of suitable habitat and abundance accelerated but uncertainties were too large to identify whether pressures acted synergistically, additively, or antagonistically. Our results suggest that conservation should prioritize the protection of suitable habitat and ensure sustainable harvesting to support a viable metapopulation under realistic levels of climate change. Inadequate management of *C. miniata* populations in the wild will likely have negative consequences for the well‐being of people relying on this ecosystem service, and we expect there may be comparable consequences relating to other medicinal plants in different parts of the world.

## INTRODUCTION

Traditional medicines are widely used in large parts of the world despite the availability of western medicine (Williams et al., 2013). It is estimated that 72% of black South Africans, in both rural and urban areas, subscribe to the use of traditional medicinal plants (Mander et al., 2007). This results in the consumption of more than 70,000 metric tons of plant material in South Africa each year, and the generation of at least 134,000 income‐earning opportunities through trade in medicinal plants and related products, which is especially important for women in low‐income communities (Williams et al., 2013). Traditional medicine users appreciate that traditional healers have a more holistic approach than modern practitioners, understanding their patient's environment better, and offering information, counseling, and treatment in a more personal manner with respect for cultural heritage (Gurib‐Fakim & Mahomoodally, 2013; Mahomoodally, 2013). Thus, the World Health Organization (WHO) encourages the consideration of traditional medicine in African member states but challenges the safety and quality of products and services, the qualification of practitioners, and the methodology and criteria for evaluating efficacy (WHO, 2013). Unfortunately, the WHO's recommendation to bring wild species of medicinal plants into cultivation systems has failed so far for a number of reasons. These include the lack of institutional support for the production and dissemination of key species for cultivation (van Wyk & Prinsloo, 2018), the long time required for many important medicinal plant species to mature (Cunningham, 1997), and the need for land area (Cunningham, 1988). Most importantly, many consumers believe that cultivation destroys the healing power of medicinal plants (Cunningham, 1993; Fennell et al., 2004). Cultivation practices can indeed alter bioactive compounds, which are typically produced to deter enemies or in response to stress in the wild (Prinsloo & Nogemane, 2018; Schippmann et al., 2002).

Consequently, wild medicinal plants are harvested in enormous quantities for personal use as well as for trade in formal and informal medicine markets, so‐called *muthi* markets (Mander et al., 2007). Traditionally, medicinal plants were exclusively harvested by trained traditional healers who respected customary conservation practices, taboos, and seasonal restrictions (van Wyk & Prinsloo, 2018; Williams et al., 2000). Today, with the involvement of profit‐oriented commercial gatherers, harvesting methods are often destructive, which leads to plant population declines (Cunningham, 1993; Williams et al., 2013). Increasing pressure from land use change will likely further contribute to medicinal plant population declines (IBPES, 2018; Lawal et al., 2019). In particular, food production contributes directly to land cover change at the local scale, which is expected to be among the main drivers of biodiversity loss in the 21st century (Pereira et al., 2010; Sintayehu, 2018). South Africa is the third‐most biologically diverse country in the world and has a complex and rapidly changing food system with both a first‐world production agricultural system and traditional harvesting of natural resources for food and other uses (Pereira & Drimie, 2016). Structural changes in food production can cause species’ habitat loss and fragmentation, isolate subpopulations, and increase the risk of local extinction in a changing climate (Opdam & Wascher, 2004). Climate change has explicitly been projected to negatively impact plant species in South Africa (Bomhard et al., 2005; Lawal et al., 2019; Midgley et al., 2002). The upward trend in temperature observed over the last century was projected to continue and extreme events such as droughts, fire, and floods will likely become more frequent (IPCC, 2014). With the loss of biodiversity, the provision of ecosystem services, including the availability of medicinal plants, could be compromised with severe consequences for human well‐being (Cardinale et al., 2012).

Although most medicinal plants are not currently threatened, special consideration in conservation policy is required to reduce future risks of regional or localized extirpation (Williams et al., 2013). Previous estimates of extents of occurrence and population declines have been based on market‐derived data or correlative species distribution models that provide only limited information on the actual longer‐term viability of a species, prompting calls for new population‐level research (Williams et al., 2013).

We answered this call for research in a case study of *Clivia miniata* (Lindl.) Verschaff. (common names are Natal lily, bush lily [English], boslelie [Afrikaans], umayime [Zulu]), one of the top 10 traded medicinal plants in *muthi* markets (Mander, 1998). The species has been reported as the most important component of a traditional healer's pallet of healing plants (Miller, 1997), which makes it subject to high harvesting pressure and depleted populations in the wild (Williams, 2005). *C. miniata* is an herbaceous evergreen flowering plant (Amaryllidaceae) that is endemic to South Africa and Swaziland. The species typically grows on steep rocky or sandy slopes in forest understory and thicket habitats (Dixon, 2011). As part of cultural and religious life, traditional healers use the roots and leaves of *C. miniata* to treat fevers, snake bites, infertility, urinary tract disorders, and to induce uterotonic activity (Musara et al., 2021; Williams et al., 2013). Antiviral and antifungal properties of alkaloids in Amaryllidaceae have been confirmed in controlled lab studies (Botting & Kuhn, 2012; Szlávik et al., 2004). *Clivia* has also been suggested as a potential candidate for HIV treatment (Rasethe et al., 2019). *C. miniata* is further of spiritual importance: unspecific parts of the plant are scattered around properties as charms against evil spirits (Miller, 1997).

The IUCN Red List categorizes *C. miniata* as “vulnerable” based on estimated population declines of at least 40% over the last 90 years that are expected to continue (Williams et al., 2008). The Red List of South African plants (SANBI, 2016) lists *C. miniata* as in danger of extinction and now rarely occurring in its ecological niches. Declines are attributed to overharvesting for medicinal plant trade and horticultural acquisitions, habitat loss to commercial forest plantations, crop cultivation, urban and coastal development, and climate change. We thus hypothesized that land use change, overharvesting and climate change will all have a negative impact on suitable habitat and metapopulation size of *C. miniata* over the next 30 years (by 2050). To explore different scenarios of these drivers, we coupled Species Distribution Models (SDMs; Peterson et al., 2011) with a metapopulation model (RAMAS‐GIS6.0; Akçakaya & Root, 2013). SDMs have provided useful insights concerning potential range shifts and extinction risk due to climate and land use change (Pearson et al., 2013; Thomas et al., 2004; Thuiller et al., 2005) and have supported conservation assessments such as for the IUCN Red List (IUCN, 2019). However, because SDMs provide limited information on and longer‐term viability of a species, we coupled this type of model with a spatially explicit metapopulation model. Metapopulation models consider life‐history traits that drive population densities under changing environmental conditions and have proven useful for extinction risk assessment, conservation research, and population viability analysis (e.g., Akçakaya, 2000; Fordham et al., 2012a, 2012b; Keith et al., 2008; Pearson et al., 2014). Further, we asked whether we could identify the nature of interactions between pressures as “synergistic” (combined pressures larger than the sum of individual pressures), “additive” (combined pressures equal to the sum of individual pressures), or “antagonistic” (combined pressures smaller than the sum of individual pressures; Côté et al., 2016). Understanding species’ response to individual as well as multiple pressures is an important contribution to prioritizing and planning conservation actions (Brown et al., 2014; Wilson et al., 2006).

## MATERIAL AND METHODS

### Species distribution model

**FIGURE 1:**
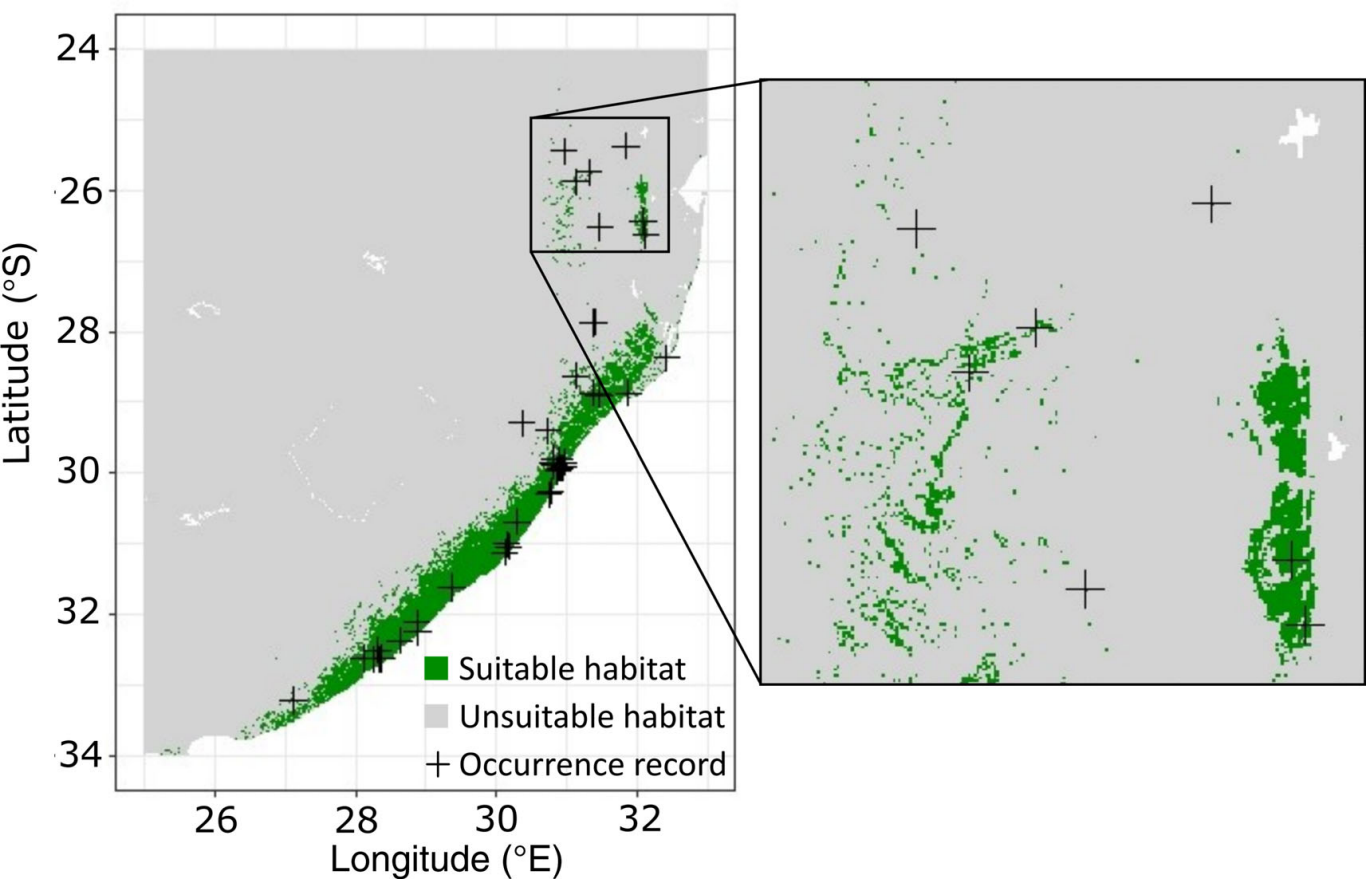
Suitable habitat (green) based on model calibration (1979–2013) and occurrence records of *Clivia miniata* (black crosses) in South Africa and Mozambique. The zoomed map on the right illustrates the small‐scale variability of habitat suitability

We built our SDM with the R package *dismo* (Hijmans et al., 2011); for details, see Appendix S1: Section S2. The model area (Figure 1) was chosen to include all occurrence records, as well as regions that experienced suitable climate during the simulation period (2020s–2050s) and that could be reached by the species, estimated with a preliminary projection of suitable habitat for the 2050s over southern Africa (south of the equator). The SDM consisted of two components: an environmental component and a land cover component. The environmental component first established a correlative relationship between bioclimatic, soil, and topographic predictors that cover the main aspects of the species’ niche (Dixon, 2011, Appendix S1: Table S2), and species occurrence data (GBIF.org, 2019) to estimate the habitat suitability for the species. We summarized model performance with the area under the curve (AUC) value of the receiver‐operating characteristic (Hanley & McNeil, 1982) and created an ensemble mean weighted by model performance (Stanton et al., 2012). To have a complementary measure of model performance, we calculated sensitivity and specificity (Lobo et al., 2008) as well as true skill statistic (TSS; Allouche et al., 2006; Table 1). Thereafter, the land cover component restricted climatically suitable habitat to areas with suitable land cover types reported for *C. miniata* (Swanevelder, 2005). We created a binary mask of suitable and unsuitable land cover types, which we multiplied with the habitat suitability maps to exclude grid cells with unsuitable land cover types. We calibrated the model to the period 1979–2013 and projected habitat suitability for each year between 2015 and 2055 and for each climate scenario that we describe. The resulting habitat suitability maps were used as inputs for the metapopulation model.

**TABLE 1:** Sensitivity, specificity, area under the curve statistics (AUC), and true skill statistic (TSS) of fivefold partitioning of a species distribution model for *Clivia miniata*

Metric and Algorithm	*k*1	*k*2	*k*3	*k*4	*k*5
Sensitivity					
MAXENT	0.56	1	1	1	1
GLM	0.889	1	1	1	1
RANDOM FOREST	1	1	1	1	1
Specificity					
MAXENT	0.4	0.48	0	0.25	0.38
GLM	0.06	0.46	0.44	0.15	0.4
RANDOM FOREST	0.12	0.57	0.08	0.11	0.12
AUC					
MAXENT	0.56	0.70	0.71	0.70	0.70
GLM	0.57	0.69	0.73	0.56	0.58
RANDOM FOREST	0.69	0.75	0.71	0.74	0.78
TSS					
MAXENT	−0.04	0.48	0	0.25	0.38
GLM	−0.05	0.46	0.44	0.44	0.4
RANDOM FOREST	0.12	0.57	0.08	0.11	0.12

*Note*: The threshold is set to “no omission.”

### Metapopulation model

We developed a spatially explicit metapopulation model for *C. miniata* using RAMAS‐GIS6.0 (Akçakaya & Root, 2013). Because there is little published information on the demography of *C. miniata*, we based the demographic model on a species of the same family, the wild daffodil (*Narcissus pseudonarcissus*; Barkham, 1980). Similar to *C. miniata*, *N. pseudonarcissus* is a long‐lived perennial geophyte, shade tolerant with similar plant structure and reproduction, and it's alkaloids have been used for centuries for medicinal purposes (e.g., Kornienko & Evidente, 2008). Barkham (1980) recorded counts of individuals in three life stages (juvenile, subadult, adult) as well as transitions between stages, fecundities and cloning, and deaths for two sites in Cumbria. We used these data to derive a set of Leslie matrices that captured our incomplete knowledge of the demography of the wild daffodil. The data had some inconsistencies that we resolved by an error minimization procedure. The Leslie matrices had dominant eigenvalues (λ) between around 0.9 and 1.0. The models are described in detail in Appendix S1: Section S3 and parameter sensitivity analysis is presented in Appendix S1: Section S4. From this pool of matrices, we selected the 2.5th percentile and 97.5th percentile eigenvalue Leslie matrix from both sites as upper and lower boundaries as well as the median eigenvalue Leslie matrix to run RAMAS. Due to data limitations, we estimated the order of magnitude for environmental stochasticity based on previous RAMAS studies on vegetation in South Africa as 20% for fecundity and 10% for transitions (Fordham et al., 2012a, 2012b). To reduce likely truncations due to high survival rates, we imposed a negative correlation between the highest survival rate and other survival rates for each stage.

Initial abundance and initial carrying capacity were estimated from *C. miniata* observations in the wild (Swanevelder, 2005), and were scaled by the patch size and habitat suitability for each population individually. Adjacent populations were delineated by a neighborhood distance threshold of 5 km, which allowed us to consider dispersal implicitly. No living vectors for long‐distance dispersal have been observed for Amaryllidaceae (Rourke, 2002). Density dependence followed a model that reduces vital rates and fecundities of all life stages whenever density exceeds a ceiling threshold, the carrying capacity. Throughout the simulations, changes in carrying capacity reflected changes in suitable habitat (Akcakaya, 2001).

For each scenario described below, we ran 10,000 replications over 30 years for both sites, starting from a stable stage distribution. We chose this duration because it corresponds to the generation length of *C. miniata* (Williams et al., 2008), which is a common strategy for conservation planning (IUCN‐SSC Species Conservation Planning Sub‐Committee, 2017). Because landscapes usually change on a time scale slower than vegetation dynamics (Akcakaya, 2001), we changed the habitat suitability map only every 5 years (running mean over 10 years, therefore we refer to years as 2020s–2050s) based on the observation from horticulture that *C. miniata* reaches blooming size in about 5 years (Pacific Horticulture Society, 2020).

### Scenarios

We designed contrasting and fairly extreme scenarios of climate (CC), land cover (LC), and harvesting to estimate the range of effects we might observe in the future. We considered all pressures isolated and in combination. Additionally, two sets of Leslie matrices represented a decreasing (site 1) and an increasing (site 2) population trend (Figure 3).

#### Climate change scenarios

We considered two climate change scenarios from the Fifth IPCC Assessment Report (IPCC, 2014): representative concentration pathways RCP2.6 and RCP8.5 (van van Vuuren et al., 2011). RCP2.6 assumes that global annual greenhouse gas emissions peak between 2010–2020, and substantially decline thereafter, which results in a projected global mean temperature rise of 0.4° to 1.7°C by the end of the century relative to 1850. In RCP8.5, emissions continue to rise throughout the 21st century and the global mean temperature is projected to rise by 2.6° to 4.8°C.

We followed a simple pattern downscaling method after Fordham et al. (2011) to generate a time series of six bioclimatic variables at 1‐km^2^ resolution from four CMIP5 General circulation models for RCP2.6 and RCP8.5. The downscaling method is described in detail in the Appendix S1: Section S1.

#### Land cover scenarios

For the first land cover scenario, we assumed that the expansion of agricultural land and urban areas stops and farming is intensified in existing agricultural areas to meet future food demand. This is equivalent to climate change only. In the second scenario, we extrapolated recent trends in land cover change arising mostly from agricultural transformation and urbanization (around 1% per year, Jewitt et al., 2015). We assumed that cropland and urban land cover replace suitable habitat in proximity to already unsuitable cells.

#### Harvesting scenarios

The first harvesting scenario (JUV) represented the preference of traders for juvenile plants, which have a lower water content than older plants (Williams et al., 2008). RAMAS harvested a fixed number of juveniles (*n* = 5) from each population every second year, to allow some time for recovery. We set the minimum population size to allow harvest to 50 individuals under the assumption that smaller populations were more difficult to locate and were therefore visited and harvested less frequently. In the second scenario (ALL), we assumed that traders do not discriminate and demand plant material from all life stages equally. To achieve similar harvesting numbers as in scenario 1, we set RAMAS to harvest the same fixed number of individuals from all stages combined every 2 years under the same constraint of a minimum population size of 50 individuals. Note that this constraint limited harvesting at low numbers towards the end of the simulations, thus the total number of individuals harvested per scenario was not identical.

## RESULTS

### Effects on suitable habitat area

**FIGURE 2:**
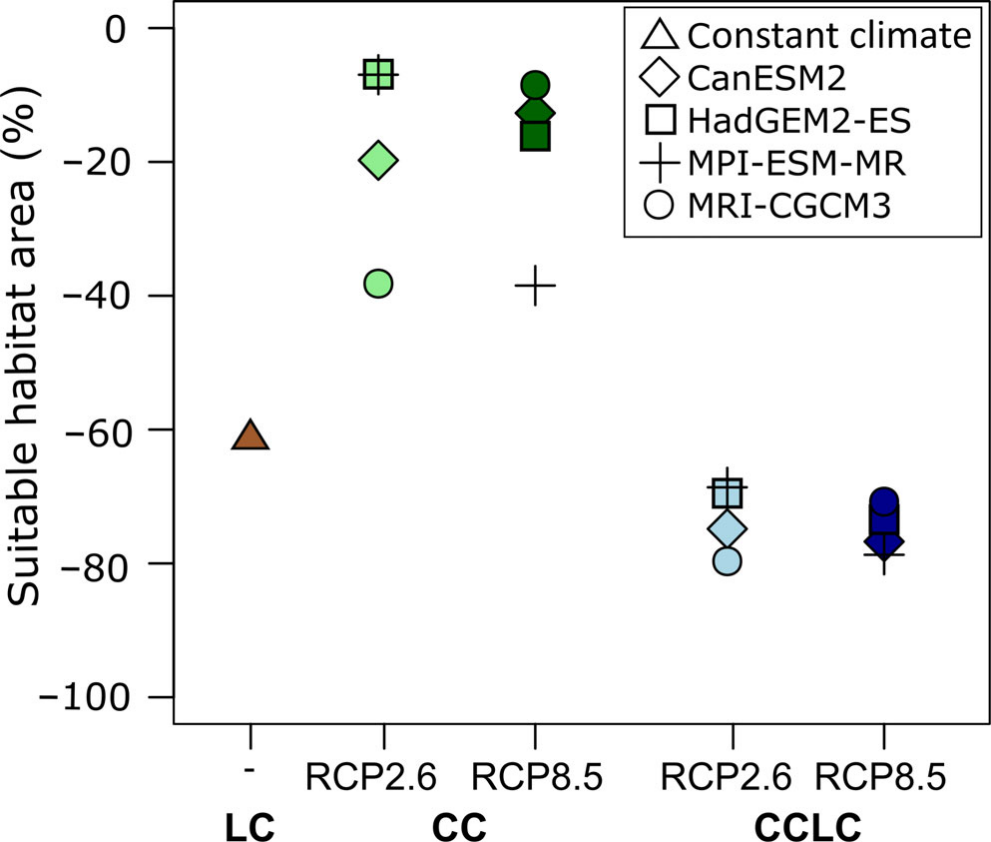
Loss of *Clivia miniata*'s suitable habitat area between the 2020s and 2050s for land cover change only (LC, brown), climate change only (CC, green), and climate and land cover change combined (CCLC, blue) under RCP2.6 and RCP8.5 for four general circulation models (see Appendix S1: Table S1 for details). Each data point represents the thresholded and weighted ensemble mean habitat suitability provided by the SDM

The suitable habitat area for *C. miniata* was projected to decline under all climate change and land cover scenarios (Figure 2). Looking at the full range of GCMs, CC reduced the mean suitable habitat area by around 14% ± 12.2% and 14.2% ± 9.6% for RCP2.6 and RCP8.5, respectively. Increasing temperatures and decreasing precipitation drove the decline in both climate scenarios. However, not all scenarios caused a monotonous decline: CanESM2 RCP2.6 and HadGEM2‐ES RCP2.6 led to an increase in suitable habitat area before a reduction of around 20% (relative to the start of the simulation) by the 2050s. LC caused substantially higher losses of suitable habitat area with more than 61% relative to the initial conditions. In combination, CCLC reduced the suitable habitat area by 71.5% ± 4.2% (RCP2.6) and 73.2% ± 2.1% (RCP8.5). Overall, land cover change seemed to be the main driver of changes in suitable habitat area.

To prioritize and plan conservation actions, it is important to understand the species’ response to individual as well as multiple pressures. We compared the sum of individual effect sizes (CC + LC) to the simulations with both pressures (CCLC) to test whether the nature of interactions between land cover and climate change was synergistic, additive, or antagonistic. Looking at individual GCMs, we observed three antagonistic interactions, four synergistic interactions, and one additive interaction (Figure 2, Appendix S1: Table S14). However, when considering the mean of the model ensemble, the signal moderated and the range of uncertainty was too large to detect a distinct type of interaction.

### Effects on abundance

**FIGURE 3:**
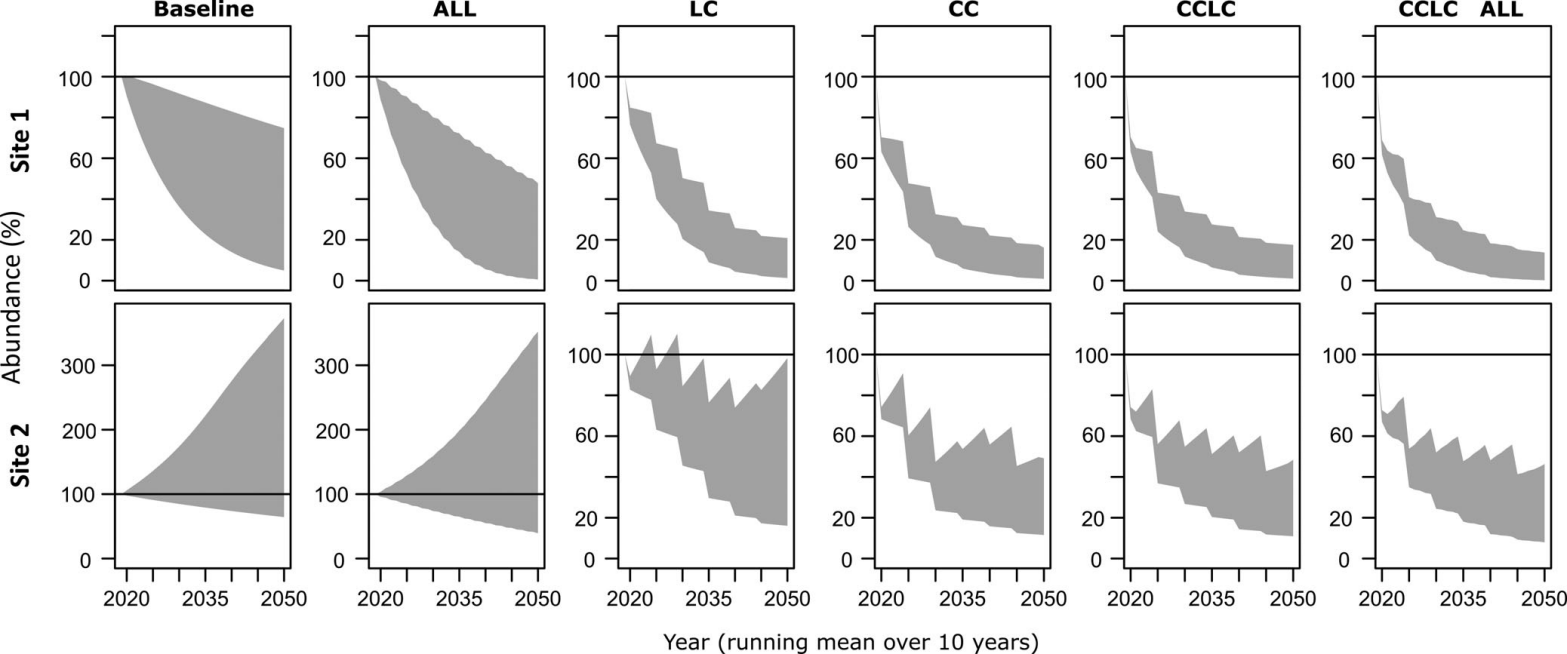
Abundance of *Clivia miniata* between 2020s and 2050s (in percentage of initial conditions) for the baseline scenario, harvesting all life stages only (ALL), land cover change only (LC), climate change only (CC, CanESM2 RCP2.6), climate and land cover change (CCLC, CanESM2 RCP2.6), and climate and land cover change with harvesting from all life stages (CCLC ALL, CanESM2 RCP2.6). The gray‐shaded areas depict the 95th percentiles of eigenvalues sampled from Barkham (1980) for site 1 (top row) and site 2 (bottom row). Note the altered scale for the baseline and ALL scenario at site 2

Similar to the suitable habitat area, the abundance of *C. miniata* was projected to decline. In relation to the change in suitable habitat area, we observed a disproportionally strong decline in abundance. Compared to the baseline scenario, the negative population trend based on site 1 observations was further reduced by environmental pressures, and the positive population trend based on site 2 observations was reversed into a negative trend (Figure 3). The only exception was the harvesting‐only scenario, which did not change the trend but capped the potential population growth. This was likely an artifact of the model set up which constraint harvesting to populations larger than 50 individuals, which were much more common when the metapopulation was not diminished by environmental pressures.

Because the trajectories of all GCMs had a similar shape, we illustrate the temporal changes in abundance (in percent, relative to the initial conditions) using the example of CanESM2 RCP2.6 (Figure 3). In all scenarios, the 95th percentile shifted toward lower values, indicating an overall negative change in abundance. Uncertainty increased over time in all scenarios but decreased with the addition of pressures. The baseline scenario had clearly the largest uncertainty range at the end of the simulations with around 5% to 75% (site 1) and 65% to 370% (site 2). Harvesting adults from the baseline affected mostly the upper end of the distribution showing uncertainty ranges of 1% to 48% (site 1) and 39% to 351% (site 2). The other scenarios caused stronger population declines accompanied by reductions in uncertainties. By the 2050s, the abundance was projected to decline to around 1% to 20% (site 1) and 20% to 100% (site 2) for LC, and around 1% to 20% (site 1) and 10% and 50% (site 2) for CC, CCLC, and CCLC ALL. Apart from harvesting, any pressure considered could push the metapopulation to high extinction risk in our model, especially if the population behaved according to site 1 dynamics.

**FIGURE 4:**
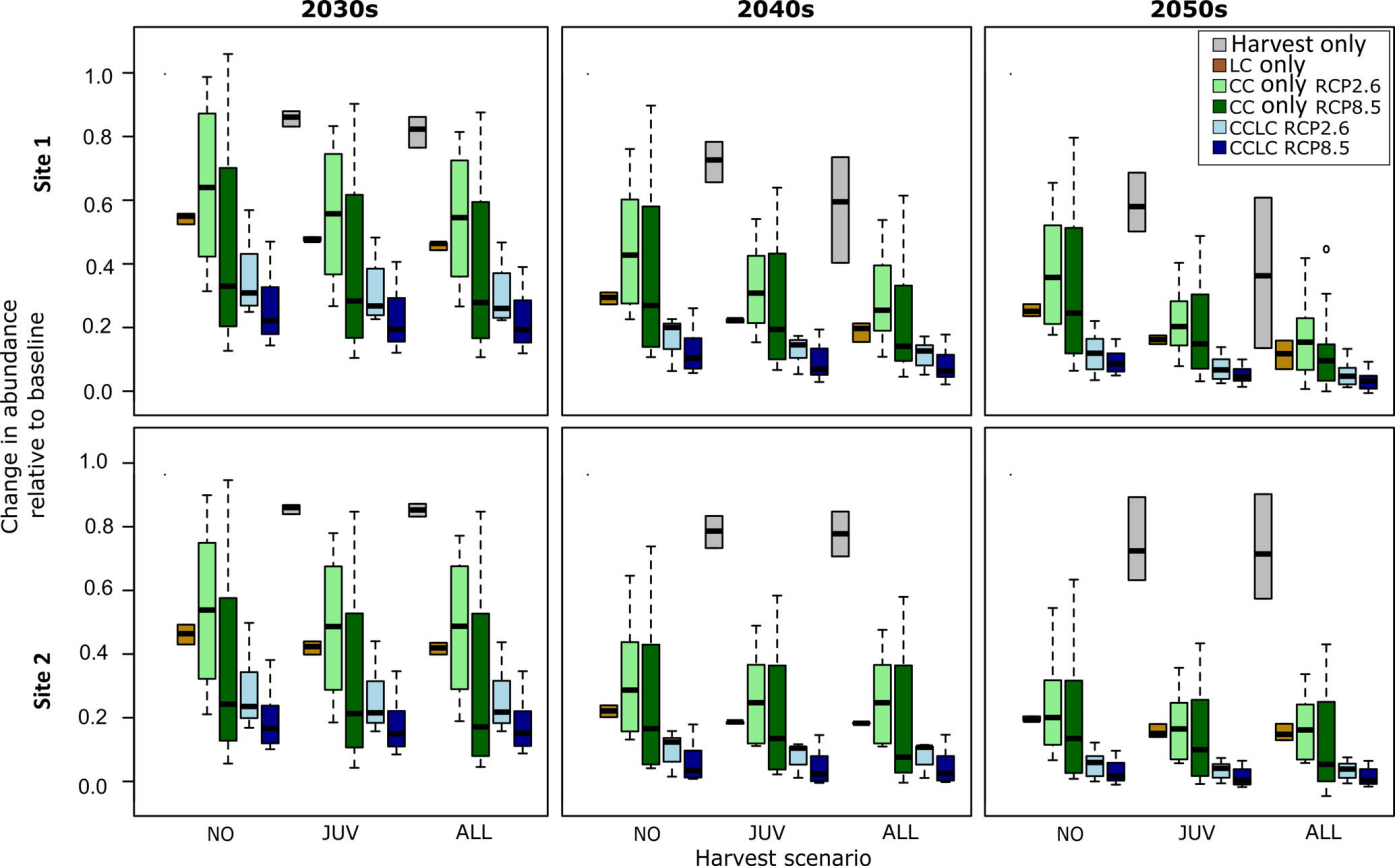
Changes in abundance of *Clivia miniata* since the beginning of the simulation (2020s) relative to the baseline scenarios for 2030s, 2040s, and 2050s for harvesting only (gray), land cover change only (LC, brown), climate change only (CC, light green for RCP2.6, dark green for RCP8.5), climate and land cover change (CCLC, light blue for RCP2.6, dark blue for RCP8.5), and three harvesting scenarios (no harvest [NO], harvest only juveniles [JUV], harvest from all stages [ALL]). Each box contains 12 results (four GCMs and three Leslie matrices), except LC, which includes only three data points (three Leslie matrices). Boxplot components are mid line, median; box edges, interquartile range; and whiskers, highest and lowest values

To compare the effects of individually acting and combined pressures, we summarized changes relative to the baseline of all simulations in Figure 4. Each box contains 12 results (four GCMs, and three Leslie matrices), except LC and harvesting‐only scenarios, which include only three data points (three Leslie matrices). The full range of simulations showed clear differences between scenarios despite the large uncertainty ranges. The effect sizes of LC and CC could not be distinguished; however, the combined effect CCLC seemed to be always stronger than the individual effects. The difference between RCP2.6 and RCP8.5 was not significant, but the median of RCP8.5 always lied below RCP2.6. Similarly, there was no significant difference between CC and CCLC, although CCLC consistently fell below CC. Harvesting pressure in isolation had the smallest effect due to the model‐setting artifacts we discussed. However, in accordance with our hypothesis and life history theory, harvesting from all stages resulted in a faster decline in abundance than extracting only juveniles. With the present model set up, we could not find sustainable harvesting levels under CC or CCLC. The addition of pressures increased the negative impact on the metapopulation, but the large uncertainties do not allow us to conclude the nature of the interaction. Comparing the two sites showed that the relative effect was independent of the direction of baseline population trend, once more highlighting that the external effects of environmental change could override internal population dynamics.

## DISCUSSION

Our results concur with previous concerns about continuing trends of already declining *C. miniata* populations under climate change, land cover change, and harvesting pressures (Williams et al., 2008) and supported our hypothesis. Overall, external effects of environmental change overrode internal population dynamics in our study. Considering pressures independently, the future loss of suitable habitat was mainly driven by land cover change, which is methodologically coherent and ecologically reasonable: once land cover has changed, land is usually permanently lost to the species. This is in line with previous studies that established land cover change as a major threat to biodiversity over the next decades (Jewitt et al., 2015; Pereira et al., 2010). Whereas land cover change had irreversible consequences for the suitable habitat, climate suitability fluctuated between years, and unsuitable areas could become suitable again. Because populations do not go extinct after one “bad” year, *C. miniata* was less affected by climate change in our simulations, and will, therefore, probably be more resilient to climate variability than to land cover change over the next decades. This might change beyond 2050 as climate change is expected to become a more important threat to biodiversity (Leadley et al., 2010; Newbold, 2018) due to continuous upward trends in temperature and an increased frequency of extreme events such as droughts, fire and floods (Jentsch et al., 2007; Oliver & Morecroft, 2014). In combination, land cover and climate change increased the effects on the suitable area, but the range of uncertainty was too large to detect whether the interaction between variables was synergistic, additive, or antagonistic. Future research could explore the type of interaction further; for example, synergistic interactions could occur if habitat loss were to concentrate harvest into smaller areas, causing the harvest to exceeded maximum sustainable levels.

Remarkably, the trends in suitable area did not translate linearly into changes in abundance in our simulations. Such a disproportional relationship has been previously reported for vegetation in RAMAS (Fordham et al., 2012a, 2012b, 2013; Swab et al., 2015) and other systems (Newbold et al., 2020). The results confirm that the extent of suitable area, as well as the metapopulation structure and its interaction with the demography, are crucial for metapopulation trends (Oliver & Morecroft, 2014; Opdam, 1991). This is particularly relevant in combination with climate‐induced range shifts (Oliver et al., 2013; Opdam & Wascher, 2004; Piessens et al., 2009). As for suitable area, the range of uncertainty in abundances was too large to detect a distinct type of interaction.

Whereas climate and land cover change are expected to accelerate in future decades (IPCC, 2014), overharvesting is perhaps the most concerning threat to *C. miniata* in the short term (Williams, 2005). Harvesting was not the dominant pressure in our model, partly because we did not have accurate quantitative estimates of initial metapopulation size and harvest quantities. The relative contribution of harvesting eventually depends on the absolute quantity, as well as the frequency and timing of harvesting events (Ghimire et al., 2008; Ticktin, 2004), which goes beyond the scope of this study. However, we can conclude that harvesting from all stages resulted in a stronger decline in abundance than extracting only juveniles, as predicted from life history theory and shown in previous studies on herbaceous perennial plants (Rock et al., 2004; Schemske et al., 1994). If the demand for *C. miniata* products remains high or even increases in the future as viral disease are more likely to occur (Settele et al., 2020), wild populations could be harvested unsustainably and irrespective of size. An increase in medicinal plant use has been documented during the Covid‐19 pandemic in 2020 (Khadka et al., 2021) and research focused quickly on the identification of potential SARS‐CoV‐2 inhibitors from medicinal plant extracts (ACEDHARS UNILAG COVID‐19 Response Team, 2020; Dwarka et al., 2020). Insufficient supply of *C. miniata* products will not only affect the health care of a large part of the South African population and diminish income earning opportunities for low‐income households. It will also compromise the execution of traditional ceremonies and rituals, which are an irreplaceable cultural asset and heritage. In combination with climate and land cover change, harvesting acted as another worsening effect on the abundance of *C. miniata*. Similarly, Mora et al. (2007) showed that interacting effects between overexploitation and fragmentation could reduce the resistance of populations to climate change. We want to emphasize uncertainties in our study due to limited data availability and quality, as well as model and scenario selection, discussed in detail in Appendix S1: Section S6. Yet despite these limitations, our case study is typical in terms of the limited data that is available for species of conservation interest, and our work thus provides an important benchmark as to what can, and cannot, be achieved using current practice modeling methods.

### Conservation priorities and future directions

We deduce that successful conservation of *C. miniata* will require actions that minimize all three pressures: climate change, land cover change, and overharvesting. It is apparent that projected land cover change is an immediate threat to this species’ suitable habitat, thus securing high quality “source” populations in good quality habitat is a priority. This could be achieved through legal regulation on land conversion and the protection of habitat in the form of conservation areas. Given that climate change adds to the risks from land cover change, and that there are limited actions at site scale that address climate change, more research should be focused on the climate sensitivity of the species and potential relocation areas. Harvesting regulations should prioritize the protection of established individuals to allow faster recovery of threatened populations while ensuring the achievement of sustainable harvesting. The potential for cultivation, commercially and on household scale, should be re‐evaluated. It is also essential to increase harvester and healer's awareness to perceive the eventual problems and solutions.

Looking beyond medicinal plants, the method presented here can be applied to other species that supply ecosystem services such as edible indigenous plants that are harvested from the wild, pollinating insects, or pest controlling spiders. With the support of additional observational data, metapopulation models could provide valuable input to the IUCN Red List evaluation process. Future studies could explore a range of environmental and socioeconomic scenarios to inform conservation decision making. If land cover change focused on areas transformed for food production, diet‐driven scenarios (omnivore vs. vegetarian) could be explored. The report on Land Reform Futures provides one example of socioeconomic scenarios for South Africa (Vumelana Advisory Fund, 2016). This report illustrates four possible futures based on power distribution and land ownership. Translating these scenarios into spatial land cover maps for future studies requires interdisciplinary cooperation with different stakeholders.

## CONFLICT OF INTEREST

The authors declare no competing interests.

## AUTHOR CONTRIBUTIONS

Our study brings together researchers from different countries, including researchers based in the country where the study was performed. Vivienne P. Groner, Georgina M. Mace, and Richard G. Pearson conceptualized the study; Vivienne P. Groner and Owen Nicholas carried out the simulations and analysis; H. Reşit Akçakaya advised on methodology and software; and Vivienne P. Groner led paper writing with input from all authors.

## Supporting information


Appendix S1

## Data Availability

Data used in this study are as follows. Climate data: Karger et al., 2017a, 2017b; CHELSA, 2019; ESGF, 2019. Land cover data: Broxton et al., 2014. Predictors for the species distribution model: Amatulli et al., 2018; Fischer et al., 2008; GBIF.org, 2019. Additional references for occurrence records used to create background points are provided in Appendix S1: Table S3. Code (vrgo, 2021) is available on Zenodo at https://doi.org/10.5281/zenodo.5342210.
