# Supplementary material for: Climate change, land cover change, and overharvesting threaten a widely used medicinal plant in South Africa
Source: Ecol Appl. 2022 Mar 21;32(4):e2545. doi: 10.1002/eap.2545 (PMC9286539; doi:10.1002/eap.2545)
Supplement: Supplementary file 1 — Appendix S1 [file EAP-32-0-s001.pdf]

Appendix S1 for 'Climate change, land cover change, and overharvesting threaten a widely used medicinal plant in South Africa'

Vivienne P. Groner, Owen Nicholas, Tafadzwanashe Mabhaudhi, Rob Slotow, H. Reşit Akçakaya, Georgina M. Mace, and Richard G. Pearson

Ecological Applications

Section S1 Climate data downscaling approach

Investigating the effects of climate change on biodiversity with a coupled species distribution - demographic model (SDM-DM) requires bioclimatic data on an ecologically relevant spatial resolution (1 to 5 km; (Seo et al. 2009)). To study the dynamics of metapopulations, this data needs to be available for every year of the considered time span. However, such a data set was not available for Southern Africa when we conducted this study. Future scenarios for climate change from the Climate Model Intercomparison Project CMIP5 (Taylor, Stouffer, and Meehl 2011) are provided on a scale of 1 to 3°, which corresponds to a grid size of 100 to 300 km. Online platforms such as http://worldclim.org (Fick and Hijmans 2017; "Worldclim" 2019) and http://chelsa-climate.org (Karger et al. 2017a; 2017b; CHELSA 2019) provide only climatologies of bioclimatic variables for the future (2050 and 2070) based on CMIP5 in 1 km resolution, but no annual time series. We followed a simple downscaling method (Fordham, Wigley, and Brook 2011) to generate a time series (2006-2099) of six bioclimatic variables (Figure S22, first column) in 1 km resolution from four CMIP5 General circulation models (Table S1) for the two representative concentration pathways RCP2.6 and RCP8.5 (van Vuuren et al. 2011) with the spatial extent -35 to 0° N, 9 to 43° E (land surface only).

Section S1.1 Model and variable selection

As a baseline for the high-resolution data set, we downloaded climatologies (1979-2013) of six bioclimatic variables freely available from http://chelsa-climate.org (Karger et al. 2017a; 2017b; CHELSA 2019), Figure S2 (first column). Evidence from various areas of numerical modelling suggests that multi-model averages often yield better, more robust predictions than a single model (Johnson and Omland 2004; Fordham, Wigley, and Brook 2011). Pierce et al. (2009) demonstrate that "With fewer than five models the results are more sensitive to the number and choice of models, while for more than five models the additional models have a relatively smaller effect on the average", (Fordham, Wigley, and Brook 2011). To account for model uncertainty, we selected four CMIP5 General Circulation Models (GCMs) represented in the 5th IPCC Assessment Report (IPCC 2014) that have previously been shown to reasonably reproduce recent climates at a global scale as well as seasonal cycle of temperature and precipitation in South Africa (McSweeney et al. 2015): CanESM2, HadGEM2-ES, MPI-ESM-MR, MRI-CGCM3, see Table S1. The GCM output is freely available to

download from the CMIP5 archive https://esgf-node.llnl.gov/projects/cmip5/ (World Climate Research Programme) ("ESGF" 2019). We downloaded the following three variables in daily resolution for part of the historical period (1979-2005) and RCP2.6/RCP8.5 (2006-2099) (ensemble r1i1p1):

- **precipitation** (variable *pr* [kg $m^2 s^{-1}$]; precipitation at surface; includes both liquid and solid phases from all types of clouds - both large-scale and convective),
- **near-surface air temperature** (variable *tas* [K]; near-surface air temperature at the 2 m height),
- **minimum/maximum near-surface air temperature** (variables *tasmin/tasmax* [K]; daily maximum/minimum near-surface air temperature at the 2 m height).

Table S1: Four General Circulation Models (GCMs) selected for this study

Modelling centre or group	Model name	Atmospheric grid	
		latitude	longitude
Canadian Centre for Climate Modelling and Analysis	CanESM2	2.7906	2.8125
Met Office Hadley Centre (UK), add. HadGEM2-ES contrib. by Inst. Nacional de Pesquisas Espaciais	HadGEM2-ES	1.25	1.875
Max Planck Institute for Meteorology (Germany)	MPI-ESM-MR	1.8653	1.875
Meteorological Research Institute (Japan)	MRI-CGCM3	1.12148	1.125

Section S1.2 Downscaling method

"The simplest method of producing high-resolution data from GCM output is the "change factor" method, where the low-resolution change from a GCM is added directly to a high-resolution baseline of observed climatology", (Fordham, Wigley, and Brook 2011). The advantage of using only GCM change data it that possible errors due to biases in the GCM's present-day baseline are avoided. Although not strictly 'downscaling' such as dynamic or statistical downscaling techniques, it provides reasonable high-resolution data to drive ecological models. Figure S1 schematically illustrates the main steps of this method. We performed the following steps with a combination of 'climate data operators'(Schulzweida 2019) and the R3.5 software. We cropped all data to Southern Africa (-35 to 0° N, 9 to 43° E, which corresponds to 4200 x 4080 grid cells at 1 km resolution) to minimize computational costs.

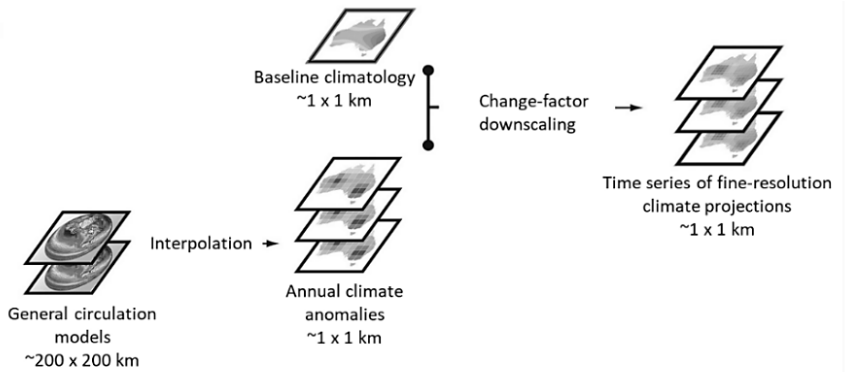

Figure S1: Change-factor downscaling method in graphic representation, modified after (Fordham, Wigley, and Brook 2011).

First, we calculated climatologies of all bioclimatic variables and GCMs (Table S1) in the original low resolution for the same period as the high resolution climatologies from CHELSA (1979-2005 from historical experiments and 2006-2013 from RCP8.5 experiments). Figure S2 highlights the differences between the CHELSA climatologies and the CMIP5 climatologies. In line with previous studies, most models overestimate precipitation and underestimate temperatures in the climatological mean (McSweeney et al. 2015). Second, we calculated an annual time series (2006-2099) of bioclimatic variables for both future scenarios (RCP2.6/RCP8.5), also in low resolution. Third, we calculated GCM climate anomalies relative to the GCM climatology in low resolution. Fourth, we added the low resolution GCM climate anomalies to the high resolution climatologies for bioclimatic variables from CHELSA. We applied bilinear interpolation to reduce discontinuities at the GCM grid cell boundaries arising from the downscaling method. Note that the data is not normalized for climate sensitivity and averaged to one multi-model average time series as described in (Fordham, Wigley, and Brook 2011).

Section S1.3 Limitations

Like every downscaling method, the “change factor” method has limitations and shortcomings. However, (Fordham, Wigley, and Brook 2011) suggest that these errors are almost certainly less than errors in the original GCM data. First, the method assumes that the fine-scale spatial variance in the climate (as characterized by the high-resolution baseline) remains unchanged over time and the relative climate change is ‘correct’. However, “Physical and statistical techniques for downscaling GCM data are either computationally demanding (as for the use of regional climate models [RCMs], and often not available for a given region) or require extensive calibration (as for statistical downscaling) and are restricted in their application to specific climate models. In both instances there are large uncertainties that make it difficult, in many cases, to justify the effort involved”, (Fordham, Wigley, and Brook 2011). Second, the method introduces errors at the GCM grid cell boundaries as well as in the grid cells that are composed of land and ocean grid cells in the high-resolution data set.

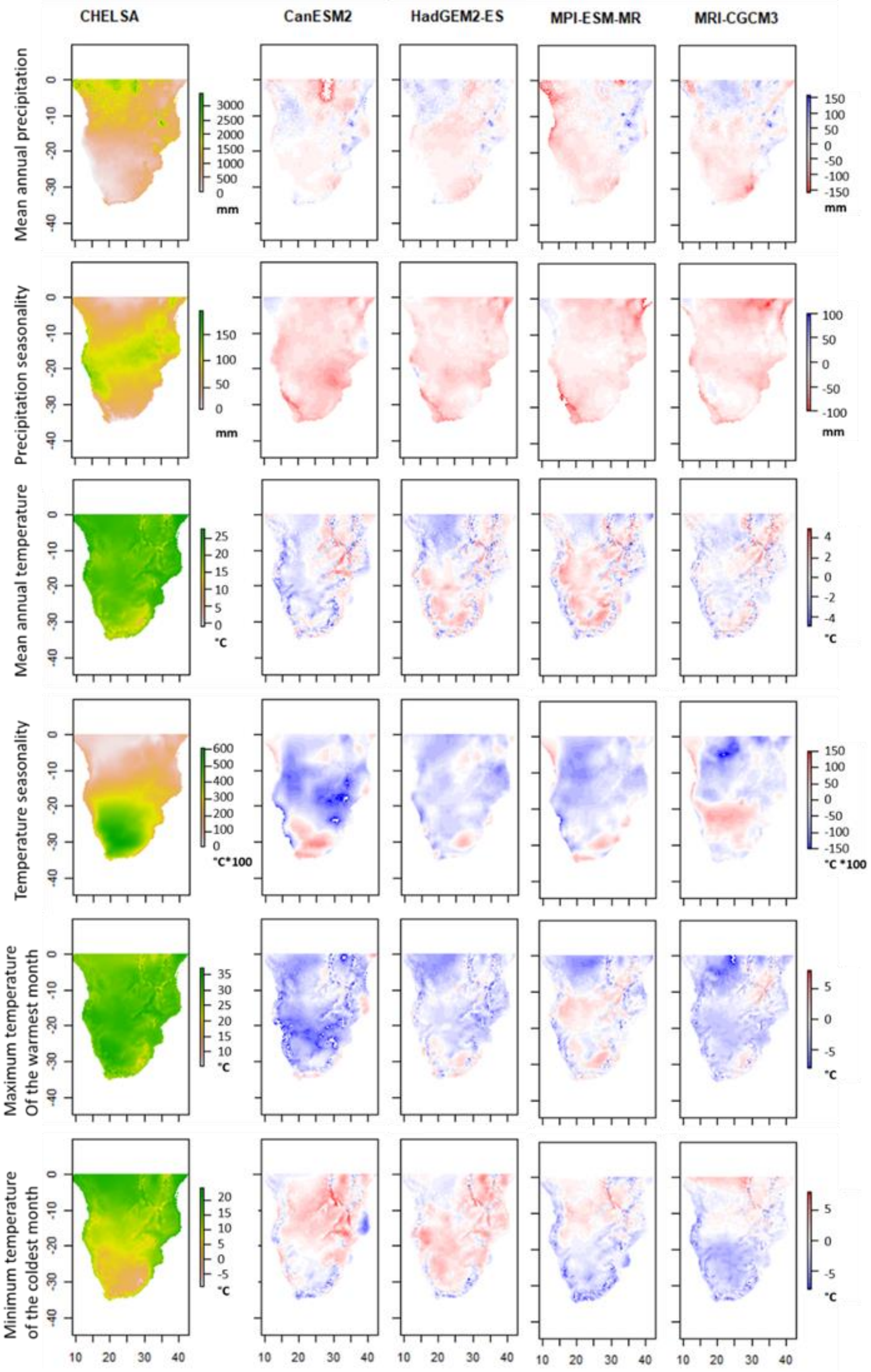

Figure S2: Differences between the CHELSA climatologies and the CMIP5 climatologies (both 1979-2013). Note that the colour bars are reversed for precipitation.

Section S2 Additional information on the species distribution model

Section S2.1 Species occurrence data

We downloaded georeferenced occurrence records for *C. miniata* from the Global Biodiversity Information Facility (GBIF.org 2019). We excluded records from before 1990 to minimize the discrepancy between occurrence records and land cover data, leaving 43 unique occurrence records with the spatial extent 24 to 34° S, 25 to 33° E, to build the SDM.

Section S2.2 Environmental data

We selected bioclimatic variables and environmental predictors that cover the main aspects of the species' niche (Tab. S2). *C. miniata*'s phenology is well adapted to an overall arid climate with bimodal rainfall that peaks in late spring an early autumn (Dixon 2011). Therefore, we chose the mean annual temperature and precipitation, as well as seasonality, as predictors. Temperature extrema were included to account for frost intolerance and a potentially higher frequency of extreme heat under climate change (Jentsch, Kreyling, and Beierkuhnlein 2007). Further, we included slope and aspect because Clivia has been reported to grow on steep slopes (Dixon 2011), preferably north-facing (RHS 2021). *C. miniata*'s distribution is associated with sandstones and quartz-rich rocks and soils (Dixon 2011), which is why we included topsoil sand content as a predictor. Climatologies (1979-2013) of all variables were downloaded at 30 arcseconds ($1km^2$) resolution from the CHELSA database (Karger et al. 2017a, b). Land cover types were used to refine the suitable area predicted by the SDM (Stanton et al. 2012). The land cover data were derived from 0.5^2 km MODIS-based Global Land Cover Climatology (Broxton et al. 2014) and scaled to 1 km^2 by bilinear interpolation (Tab. S4).

Section S2.3 Species distribution model

We built our SDM with the R package 'dismo' (Hijmans et al. 2011). The model area was chosen to include all occurrence records, as well as regions that experienced suitable climate during the simulation period (2020s-2050s) and that could be reached by the species, estimated with a preliminary projection of suitable habitat for the 2050s over southern Africa (south of the equator). The SDM consisted of two components: an environmental component and a land cover component. The environmental component first established a correlative relationship between bioclimatic, soil, and topographic predictors, and species occurrence data to estimate the habitat suitability for the species. We used three different model algorithms (generalized linear model (GLM), MAXENT, and random forest (RF)) as it has been shown that ensembles perform better than a single model (Araújo and New 2007). To account for sampling bias, we used the target-group background method of Phillips et al. (2009). Background data were selected based on occurrences of species from the same region that are included in the 'Red list of medicinal plants' (Williams et al. 2013; Tab. S3). We implemented

a cross-validation with a 5-fold partitioning procedure to define the calibration and evaluation datasets (Tab. S5). We summarized model performance with the area under the curve (AUC) value of the receiver-operating characteristic (Hanley and McNeil 1982) and created an ensemble mean weighted by model performance (Stanton et al. 2012). To have a complementary measure of model performance, we calculated sensitivity and specificity (Lobo, Jiménez-Valverde, and Real 2008) as well as true skill statistic (TSS) (Allouche, Tsoar, and Kadmon 2006). Thereafter, the land cover component restricted climatically suitable habitat to areas with suitable land cover types reported for *C. miniata* (Swanevelder 2005). We created a binary mask of suitable and unsuitable land cover types, which we multiplied with the habitat suitability maps to exclude grid cells with unsuitable land cover types, as well as grid cells classified as 'water', 'cropland' or 'urban'.

We calibrated the model to the period 1979-2013 and projected habitat suitability for each year between 2015 and 2055 and for each climate scenario described below. To create a binary habitat/non-habitat raster, we set a threshold of 'no omission', which gave model projections that included all known occurrences within the minimum area. The resulting habitat suitability maps were used as inputs for the metapopulation model.

Table S2: Bioclimatic and environmental predictor pool.

**Predictor**	**Reference**
**Mean annual precipitation**	CHELSA database (Karger et al. 2017a, b)
**Precipitation seasonality**	CHELSA database (Karger et al. 2017a ,b)
**Mean annual temperature**	CHELSA database (Karger et al. 2017a, b)
**Temperature seasonality**	CHELSA database (Karger et al. 2017a, b)
**Minimum temperature of the coldest month**	CHELSA database (Karger et al. 2017a, b)
**Maximum temperature of the warmest month**	CHELSA database (Karger et al. 2017a, b)
**Slope**	EarthEnv (Amatulli et al. 2018)
**Northness aspect**	EarthEnv (Amatulli et al. 2018)
**Topsoil sand content**	HWSD (Fischer et al. 2008)

Table S3: Species list to create background data set for Clivia miniata species distribution models.

**Adenia gummifera var. gummifera**	**GBIF.org (05 March 2019) GBIF Occurrence Download https://doi.org/10.15468/dl.4oxhyl**
**Alberta magna**	GBIF.org (27 May 2020) GBIF Occurrence Download https://doi.org/10.15468/dl.555ntr
**Aloe thraskii**	GBIF.org (27 May 2020) GBIF Occurrence Download https://doi.org/10.15468/dl.6zwa38
**Anemone fannii**	GBIF.org (05 March 2019) GBIF Occurrence Download https://doi.org/10.15468/dl.wzpzcc
**Ansellia africana**	GBIF.org (27 May 2020) GBIF Occurrence Download https://doi.org/10.15468/dl.48k5yq
**Begonia dregei**	GBIF.org (05 March 2019) GBIF Occurrence Download https://doi.org/10.15468/dl.kzufio
**Boophone disticha**	GBIF.org (27 May 2020) GBIF Occurrence Download https://doi.org/10.15468/dl.44g45h
**Bowiea volubilis subsp. volubilis**	GBIF.org (27 May 2020) GBIF Occurrence Download https://doi.org/10.15468/dl.kq96hs

**Callilepis leptophylla**	GBIF.org (05 March 2019) GBIF Occurrence Download https://doi.org/10.15468/dl.rbwvfp
**Cassipourea gummiflua var. verticillata**	GBIF.org (27 May 2020) GBIF Occurrence Download https://doi.org/10.15468/dl.ry7whb
**Cassipourea malosana**	GBIF.org (05 March 2019) GBIF Occurrence Download https://doi.org/10.15468/dl.g5fpwp
**Crinum bulbispermum**	GBIF.org (02 May 2019) GBIF Occurrence Download https://doi.org/10.15468/dl.urxvoj
**Crinum moorei**	GBIF.org (05 March 2019) GBIF Occurrence Download https://doi.org/10.15468/dl.5jgfmy
**Crinum stuhlmannii**	GBIF.org (05 March 2019) GBIF Occurrence Download https://doi.org/10.15468/dl.esal6g
**Curtisia dentata**	GBIF.org (05 March 2019) GBIF Occurrence Download https://doi.org/10.15468/dl.oumygo
**Cyrtanthus obliquus**	GBIF.org (05 March 2019) GBIF Occurrence Download https://doi.org/10.15468/dl.gk40dd
**Drimia altissima**	GBIF.org (05 March 2019) GBIF Occurrence Download https://doi.org/10.15468/dl.pvhb6x
**Elaeodendron croceum**	GBIF.org (27 May 2020) GBIF Occurrence Download https://doi.org/10.15468/dl.79jaar
**Elaeodendron transvaalense**	GBIF.org (27 May 2020) GBIF Occurrence Download https://doi.org/10.15468/dl.bwkxd3
**Encephalartos altensteinii**	GBIF.org (27 May 2020) GBIF Occurrence Download https://doi.org/10.15468/dl.zjh9nx
**Encephalartos ghellinckii**	GBIF.org (27 May 2020) GBIF Occurrence Download https://doi.org/10.15468/dl.tg76xs
**Encephalartos natalensis**	GBIF.org (27 May 2020) GBIF Occurrence Download https://doi.org/10.15468/dl.46g9ez
**Erythrophleum lasianthum**	GBIF.org (27 May 2020) GBIF Occurrence Download https://doi.org/10.15468/dl.4fpzu4
**Eucomis bicolor**	GBIF.org (27 May 2020) GBIF Occurrence Download https://doi.org/10.15468/dl.a5qg5t
**Eucomis montana**	GBIF.org (27 May 2020) GBIF Occurrence Download https://doi.org/10.15468/dl.kfx7y7
**Eulophia speciosa**	GBIF.org (27 May 2020) GBIF Occurrence Download https://doi.org/10.15468/dl.43e2cs
**Faurea macnaughtonii**	GBIF.org (27 May 2020) GBIF Occurrence Download https://doi.org/10.15468/dl.77zj3z
**Gunnera perpensa**	GBIF.org (27 May 2020) GBIF Occurrence Download https://doi.org/10.15468/dl.fsgwm2
**Hypoxis hemerocallidea**	GBIF.org (27 May 2020) GBIF Occurrence Download https://doi.org/10.15468/dl.nvdh2u
**Ilex mitis var mitis**	GBIF.org (05 March 2019) GBIF Occurrence Download https://doi.org/10.15468/dl.mfhliu
**Loxostylis alata**	GBIF.org (27 May 2020) GBIF Occurrence Download https://doi.org/10.15468/dl.3mbwzg
**Merwilla plumbea**	GBIF.org (05 March 2019) GBIF Occurrence Download https://doi.org/10.15468/dl.g36z7w
**Mondia whitei Skeels**	GBIF.org (27 May 2020) GBIF Occurrence Download https://doi.org/10.15468/dl.8vu6b9
**Newtonia hildebrandtii var. hildebrandtii**	GBIF.org (27 May 2020) GBIF Occurrence Download https://doi.org/10.15468/dl.qdmbqs
**Ocotea bullata**	GBIF.org (05 March 2019) GBIF Occurrence Download https://doi.org/10.15468/dl.4pzxge
**Prionium serratum**	GBIF.org (27 May 2020) GBIF Occurrence Download https://doi.org/10.15468/dl.c6m2rx
**Prunus africana**	GBIF.org (27 May 2020) GBIF Occurrence Download https://doi.org/10.15468/dl.ew2sbz
**Pterocelastrus rostratus**	GBIF.org (05 March 2019) GBIF Occurrence Download https://doi.org/10.15468/dl.uf0u51
**Rapanea melanophloeos**	GBIF.org (27 May 2020) GBIF Occurrence Download https://doi.org/10.15468/dl.vh4guw
**Sandersonia aurantiaca**	GBIF.org (27 May 2020) GBIF Occurrence Download https://doi.org/10.15468/dl.45xmmf
**Siphonochilus aethiopicus**	GBIF.org (27 May 2020) GBIF Occurrence Download https://doi.org/10.15468/dl.9f6zaj
**Stangeria eriopus**	GBIF.org (27 May 2020) GBIF Occurrence Download https://doi.org/10.15468/dl.tznrm5
**Warburgia salutaris**	GBIF.org (27 May 2020) GBIF Occurrence Download https://doi.org/10.15468/dl.zfvrw2

Table S4: Mask of suitable land cover types for Clivia miniata.

Land cover type	Mask
Water	0
Evergreen Needleleaf forest	1
Evergreen Broadleaf forest	1
Deciduous Needleleaf forest	1
Deciduous Broadleaf forest	1
Mixed forest	1
Closed shrublands	1
Open shrublands	1
Woody savannas	1
Savannas	1
Grasslands	0
Permanent wetlands	0
Croplands	0
Urban and built-up	0
Cropland/Natural vegetation mosaic	1
Snow and ice	0
Barren or sparsely vegetated	0

Table S5: Sensitivity, specificity, area under the curve statistics (AUC), and true skill statistic (TSS) of 5-fold partitioning of species distribution model for Clivia miniata. The threshold is set to 'no omission'.

**Algorithm**	**metric**	**k1**	**k2**	**k3**	**k4**	**k5**
	**sensitivity**					
**MAXENT**		0.56	1	1	1	1
**GLM**		0.889	1	1	1	1
**RANDOM FOREST**		1	1	1	1	1
	**specificity**					
**MAXENT**		0.4	0.48	0	0.25	0.38
**GLM**		0.06	0.46	0.44	0.15	0.4
**RANDOM FOREST**		0.12	0.57	0.08	0.11	0.12
	**AUC**					
**MAXENT**		0.56	0.70	0.71	0.70	0.70
**GLM**		0.57	0.69	0.73	0.56	0.58
**RANDOM FOREST**		0.69	0.75	0.71	0.74	0.78
	**TSS**					
**MAXENT**		-0.04	0.48	0	0.25	0.38
**GLM**		-0.05	0.46	0.44	0.44	0.4
**RANDOM FOREST**		0.12	0.57	0.08	0.11	0.12

Section S3 Additional information on metapopulation model

The RAMAS-GIS6.0 software performs three main steps. First it derives the spatial structure of the metapopulation for each year based on the projected habitat suitability maps from the SDM. A cluster of nearby grid cells with high-quality habitat is assumed to support one population. Second, RAMAS translates temporal changes in suitable habitat into changes in the mean values of carrying capacities, fecundities, and survival rates of the populations in the metapopulation, as well as the number of populations. This is implemented by 'apportioning' or 'moving' individuals among populations as they split, merge, appear, and disappear in response to changing habitat (Fig. S3). In the third step, RAMAS simulates demographic processes of death and reproduction with a stage-structured metapopulation model to calculate the expected metapopulation abundance, risk of species extinction, and other risk-based outputs.

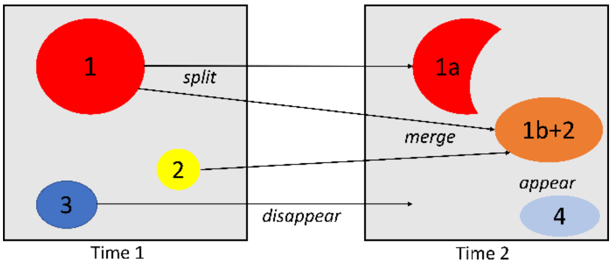

Figure S3: Habitat dynamics as implemented in RAMAS for two consecutive time steps (modified after Akcakaya 2001). At time 1, the metapopulation has three populations in three patches (1, 2, 3). In timestep two, patch 1 splits into 1a and 1b, patch 1b merges with patch 2, patch 3 disappears and patch 4 appears. Individuals from populations 1 ,2, 3 are translocated to 1a, 1b+2, 4.

Section S3.1 RAMAS input data generation

Aim and objectives

To accommodate incomplete knowledge about the demographics of *C. miniata*, the aim was here to generate representative Leslie matrices based on Wild Daffodil growth from data given by Barkham (1980) that were suitable for use with the RAMAS-GIS software. The objective was to sample potential Leslie matrices, ordered by dominant eigenvalue, and select those matrices at the 50th, 2.5th and 97.5th percentile dominant eigenvalue for use with the RAMAS-GIS software.

Source data

Barkham (1980) recorded transition and fecundity counts for two sites: from 1973 to 1978 for one site (“site 1”), and from 1973 to 1976 for the other (“site 2”). We took as source data the counts recorded in Fig. 5 of Barkham (1980), detailing flows, losses, and gains between juvenile, subadult, adult and death states.

For both sites, observations of four fecundities were given: juvenile to juvenile cloning; subadult to subadult cloning; adult to adult cloning; and adult to juvenile reproduction via seeds. Transitions between all life stages were given as well. All 12 transitions (from juvenile, subadult or adult to juvenile, subadult, adult or death) have non-zero counts recorded in Fig. 6 of Barkham (1980).

Pseudo-counts

The source data did not give complete details. For site 1, Barkham's Fig. 6 gives transition counts from juvenile to subadult or adult, but not transition counts from juvenile to subadult and juvenile to adult separately. Taking the source data at face value also presented difficulties. As an example, Fig. 6 gives that on site 2 in 1973 there are 73 juveniles, 1 of which has transitioned to a subadult by 1974. On the other hand, Barkham gives that in 1974 there are 26 subadults, 5 of which transitioned from juvenile: it appears that the number of transitions from juvenile in 1973 to subadult in 1974 is recorded as 1 in one place, and 5 in another. Thus, we generated pseudo-counts of the 12 transition counts for each year, for each site, by minimising a measure of discrepancy between Barkham's data and hypothetical counts.

Probabilistic modelling

We fitted probabilistic models for each site, using all the years' pseudo-counts to fit a single probabilistic model with coefficients that are constant in time.

For transitions, we fitted a multinomial distribution for the probability of transition from each state to any state yielding, for each site, three multinomial distributions (one for juvenile, subadult and adult) with four possibilities (transition to juvenile, subadult, adult or death). For each site we fitted a Poisson model for each of the four fecundity terms.

Sampled Leslie matrices

We generated a set of potential Leslie matrices by sampling the parameters of the transition and fecundity distributions consistent with pseudo-counts and combining transition and fecundity probabilities by addition to produce a 3 by 3 Leslie matrix (dropping transitions to death). For each site and for each Leslie matrix, we computed the dominant eigenvalue. Each site's matrices were then ordered by the dominant eigenvalue, with the 2.5th, 50th and 97.5th centile matrix chosen from each site as an input to RAMAS.

Numerical details

We set out transition information in the 4 by 3 matrix format

**Juvenile to juvenile**	**Subadult to juvenile**	**Adult to juvenile**
**Juvenile to subadult**	Subadult to subadult	Adult to subadult
**Juvenile to adult**	Subadult to adult	Adult to adult
**Juvenile to death**	Subadult to death	Adult to death

with columns corresponding to source, and rows to destination, and fecundity information in the 3 by 3 matrix format

**Juvenile to juvenile**		**Adult to juvenile**
	Subadult to subadult	
		Adult to adult

For each site and for each year, Barkham gives information about transitions and fecundities. For fecundities, this information is in terms of the four elements of the fecundity matrix.

For transitions, for site 1, information is given for 20 different combinations of transitions (up to four combinations), some of which overlap.

Table S6: Transitions for site 1 given by Barkham (1980).

	Combination of transitions				Year				
					1973	1974	1975	1976	1977
	**Juvenile numbers**								
**1**	Juvenile to juvenile				22	16	15	14	14
**2**	Juvenile to subadult	Juvenile to adult			2	1	2	1	1
**3**	Juvenile to death				4	11	2	2	2
**4**	Subadult to juvenile	Adult to juvenile			1	0	2	3	3
	**Subadult numbers**								
**5**	Subadult to juvenile				1	0	2	0	1
**6**	Subadult to subadult				22	19	12	16	17
**7**	Subadult to adult				6	6	10	1	2
**8**	Subadult to death				3	4	0	1	4
**9**	Juvenile to subadult				3	1	1	1	1
**10**	Adult to subadult				4	4	3	6	5
	**Adult numbers**								
**11**	Adult to juvenile	Adult to subadult			4	3	3	12	5
**12**	Adult to adult				24	27	28	25	21
**13**	Adult to death				3	1	1	3	0
**14**	Juvenile to adult	Subadult to adult			7	5	11	1	4
	**Row totals**								
**15**	Juvenile to juvenile	Subadult to juvenile	Adult to juvenile		23	16	17	17	17
**16**	Juvenile to subadult	Subadult to subadult	Adult to subadult		29	24	16	23	23
**17**	Juvenile to adult	Subadult to adult	Adult to adult		31	32	39	26	25
	**Column totals**								
**18**	Juvenile to juvenile	Juvenile to subadult	Juvenile to adult	Juvenile to death	28	28	19	17	17
**19**	Subadult to juvenile	Subadult to subadult	Subadult to adult	Subadult to death	32	29	24	18	24
**20**	Adult to juvenile	Adult to subadult	Adult to adult	Adult to death	31	31	32	40	26

For site 1, Barkham gives the following fecundities

Table S7: Fecundities for site 1 given by Barkham (1980).

	Year				
	1973	1974	1975	1976	1977
**Juvenile to juvenile**	1	2	0	0	2
**Subadult to subadult**	0	0	2	1	3
**Adult to adult**	0	0	1	0	0
**Adult to juvenile**	4	1	0	0	0

For site 2, information is given for 23 combinations of transitions (up to 4 combinations), some of which overlap, and some of which are identical.

Table S8: Transitions for site 2 given by Barkham (1980).

	Combination of transitions				Year		
					1973	1974	1975
	**Juvenile numbers**						
**1**	Juvenile to juvenile				52	52	56
**2**	Juvenile to subadult				1	4	6
**3**	Juvenile to death				20	52	47
**4**	Subadult to juvenile				0	2	2
**5**	Adult to juvenile				1	0	1
	**Subadult numbers**						
**6**	Subadult to juvenile				0	2	2
**7**	Subadult to subadult				9	17	18
**8**	Subadult to adult				16	5	11
**9**	Subadult to death				1	2	4
**10**	Juvenile to subadult				5	4	6
**11**	Adult to subadult				10	11	13
	**Adult numbers**						
**12**	Adult to juvenile				1	0	0
**13**	Adult to subadult				10	10	14
**14**	Adult to adult				113	123	111
**15**	Adult to death				1	5	9
**16**	Juvenile to adult				1	0	0
**17**	Subadult to adult				16	5	11
	**Row totals**						
**18**	Juvenile to juvenile	Subadult to juvenile	Adult to juvenile		53	54	59
**19**	Juvenile to subadult	Subadult to subadult	Adult to subadult		24	32	37
**20**	Juvenile to adult	Subadult to adult	Adult to adult		130	128	122
	**Column totals**						
**21**	Juvenile to juvenile	Juvenile to subadult	Juvenile to adult	Juvenile to death	73	108	109
**22**	Subadult to juvenile	Subadult to subadult	Subadult to adult	Subadult to death	26	26	35
**23**	Adult to juvenile	Adult to subadult	Adult to adult	Adult to death	125	138	134

For site 2, Barkham gives the following fecundities:

Table S9: Fecundities for site 2 given by Barkham (1980).

	Year		
	1973	1974	1975
**Juvenile to juvenile**	5	1	1
**Subadult to subadult**	2	3	1
**Adult to adult**	8	6	1
**Adult to juvenile**	50	54	12

To derive pseudo-counts for site 1 (and similarly for site 2) we created 20 4 by 3 matrices H_i, $1 \leq i \leq 20$, with elements $H_{i,jk}$ where $1 \leq j \leq 4$ and $1 \leq k \leq 3$, such that the j indices with values $1, 2, 3, 4$ correspond to juvenile, subadult, adult and death; and the k indices with values $1, 2, 3$ correspond to juvenile, subadult and adult. The H_i matrix corresponds to the ith entry in the site 1 transitions

table and has jkth entry zero unless the k to j transition is listed in the ith entry, in which case $H_{i,jk}$ is 1. Denoting by y_{il} the counts corresponding to the ith row in the table for year l, we used Powell's method to find the values $T_{l,jk}$ which minimise

$$\sum_{i=1}^{20}\left|y_{il}-\sum_{j=1,k=1}^{j=4,k=3}H_{i,jk}T_{l,jk}\right|$$

This has the effect of finding the transition "counts" $T_{l,jk}$ (with real values) which minimise the sum of absolute differences between counts and corresponding combinations of $T_{l,jk}$.

We finally rounded the entries of $T_{l,jk}$ to the closest integer to generate 12 pseudo-counts of transition counts for each year for site 1. Similarly, for site 2, with 23 counts. The resulting pseudo-counts for each site are:

Table S10: Pseudo-counts for site 1.

	Site 1 pseudo-counts					
	**Transition**	**Year**				
		1973	1974	1975	1976	1977
**1**	Juvenile to juvenile	22	16	15	14	14
**2**	Juvenile to subadult	2	1	0	1	1
**3**	Juvenile to adult	1	0	2	0	1
**4**	Juvenile to death	4	11	2	2	2
**5**	Subadult to juvenile	1	0	1	0	2
**6**	Subadult to subadult	22	19	14	16	17
**7**	Subadult to adult	6	6	9	1	3
**8**	Subadult to death	3	4	0	1	4
**9**	Adult to juvenile	0	0	1	4	1
**10**	Adult to subadult	4	4	2	6	4
**11**	Adult to adult	24	26	28	25	21
**12**	Adult to death	3	1	1	4	0

Table S11: Pseudo-counts for site 2.

	Site 2 pseudo-counts			
	**Transition**	**Year**		
		1973	1974	1975
**1**	Juvenile to juvenile	52	52	56
**2**	Juvenile to subadult	4	4	6
**3**	Juvenile to adult	1	0	0
**4**	Juvenile to death	18	52	47
**5**	Subadult to juvenile	0	2	2
**6**	Subadult to subadult	9	17	18
**7**	Subadult to adult	16	5	11
**8**	Subadult to death	1	2	4
**9**	Adult to juvenile	1	0	1
**10**	Adult to subadult	10	10	13
**11**	Adult to adult	113	123	111
**12**	Adult to death	1	5	9

Taking these pseudo-counts, we fitted separate models for each site. We fitted multinomial models for transitions

$$\{T_{l,jk}\}_{1\le j\le 4} \sim \mathrm{Multinomial}(\alpha_{k1}, \alpha_{k2}, \alpha_{k3}, \alpha_{k4})$$

and Poisson models for fecundities $F_{l,11}$, $F_{l,22}$, $F_{l,33}$ and $F_{l,13}$

$$F_{l,jk} \sim \mathrm{Poisson}(\rho_{jk})$$

We did so by sampling 7,500 sets of parameters for each site using Bayesian modelling, with uninformative priors for $\alpha_{k1} \ge 0$ and $\rho_{jk} \ge 0$. We then added the multinomial transition probabilities and the Poisson fecundity probabilities to produce 7,500 3 by 3 Leslie matrices (omitting the death state) for each site, which were ranked by their dominant eigenvalue:

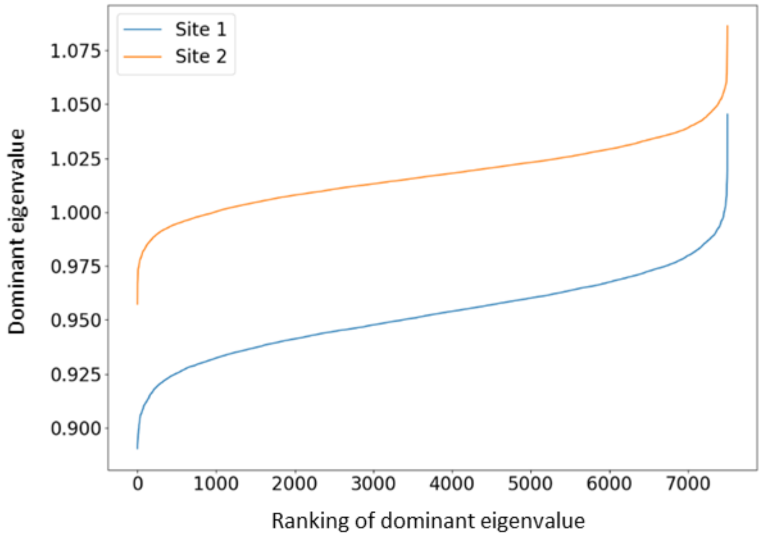

Figure S4: Ranking of dominant eigenvalues simulated with the multinominal models described here.

For each site we then took the 50th, 2.5th and 97.5th percentile Leslie matrix (in terms of dominant eigenvalue ranking). It is important to note that the adult column totals for site 2 exceed 1. This is because of site 1's high cloning rates.

Table S12: 50th, 2.5th and 97.5th percentile Leslie matrix data for site 1 and 2. As reference, data for a matrix with λ =1 (out of the 7500 matrices we simulated).

Transition+fecundity	Site 1				Site 2			
	50	2.5	97.5	$\lambda = 1$	50	2.5	97.5	$\lambda = 1$
**Juvenile to juvenile**	0.760	0.747	0.782	0.787	0.561	0.587	0.595	0.547
**Juvenile to subadult**	0.025	0.040	0.030	0.013	0.016	0.059	0.039	0.022
**Juvenile to adult**	0.085	0.063	0.103	0.034	0.327	0.337	0.303	0.304
**Subadult to juvenile**	0.035	0.075	0.065	0.041	0.050	0.046	0.046	0.058
**Subadult to subadult**	0.741	0.710	0.714	0.796	0.637	0.568	0.764	0.603
**Subadult to adult**	0.129	0.171	0.124	0.066	0.099	0.075	0.064	0.100
**Adult to juvenile**	0.061	0.014	0.054	0.050	0.005	0.001	0.008	0.001
**Adult to subadult**	0.202	0.150	0.236	0.205	0.325	0.284	0.356	0.408
**Adult to adult**	0.779	0.750	0.818	0.917	0.897	0.908	0.921	0.909

Section S3.2 Implementation in RAMAS

Barkham (1980) recorded counts of individuals in three life stages ('juvenile', 'subadult', 'adult') as well as transitions between stages, fecundities and cloning, and deaths from 1973 to 1978 for 'site 1', and from 1973 to 1976 for 'site 2'. First, we applied the least absolute deviations method on the observed numbers of individuals to account for inconsistencies in the raw published data (measurement error). With this new data set, we sampled 7,500 Leslie matrices for each site that cover dominant eigenvalues (λ) between around 0.9 and 1 using Bayesian estimation to accommodate incomplete knowledge. New individuals produced by seed or cloning (Fecundities) were taken from a Poisson distribution. We assumed no year-to-year change in the transition, fecundity, or cloning probabilities in this step. From this pool of matrices, we selected the 2.5 percentile and 97.5 percentile eigenvalue Leslie matrix from both sites as upper and lower boundaries as well as the median eigenvalue Leslie matrix to run RAMAS. Due to data limitations, we estimated the order of magnitude for environmental stochasticity based on previous RAMAS studies on vegetation in South Africa as 20 % for fecundity and 10 % for transitions (Fordham et al. 2012). Sensitivity studies on year-to-year variability showed that in comparison with the range of uncertainty about the actual Leslie matrix, variations in standard deviations made very little difference to the simulations (Tab. S13). To reduce likely truncations due to high survival rates, we imposed a negative correlation between the highest survival rate and other survival rates for each stage (Akçakaya & Root, 2005).

Initial abundance and initial carrying capacity were estimated from C. miniata observations in the wild (Swanevelder 2005) and were scaled by the patch size and habitat suitability for each population individually. Adjacent populations were delineated by a neighbourhood distance threshold of 5 km, which allowed us to consider dispersal implicitly. No living vectors for long-distance dispersal have been observed for Amaryllidaceae (Rourke 2002). Bird dispersal usually occurs between closely adjacent forest patches, while dispersal by monkeys is reported to occur close to the parent plant and is generally limited to tens of meters (Kiepiel and Johnson 2019). Because the distance between model patches was larger than the observed seed dispersal by rodents, birds, or primates, we assumed that grid cells that become suitable within the range of dispersal are included in the existing populations. Density dependence followed a model that reduces vital rates and fecundities of all life stages whenever density exceeds a ceiling threshold, the carrying capacity (Keith et al. 2008). Throughout the simulations, changes in carrying capacity reflected changes in suitable habitat (Akcakaya 2001).

Section S4 RAMAS Sensitivity analysis

Because of limited data availability, RAMAS parameter were difficult to estimate without large uncertainties. To get an idea of the effects of different parameter values, we tested a range of values for each of the following parameters at the example of 'CanESM2 RCP2.6, climate change only, no harvest': initial abundance and initial carrying capacity, time step for change in habitat suitability, neighborhood distance, Leslie matrix standard deviations, and dispersal. The sensitivity analysis is described in the following and the main results are summarized in Tab. S13.

Section S4.1 Initial abundance and carrying capacity

Initial abundance (IA) and initial carrying capacity (ICC) were estimated from *C. miniata* observations in the wild (Swanevelder 2005) and implemented in RAMAS as a function of patch size (number of cells, 'noc') and absolute habitat suitability ('ahs') in the patch. To prevent unrealistically high numbers of individuals in the largest patches, which leads to errors in RAMAS, we chose a logarithmic function for IA. To test the sensitivity to initial abundance and carrying capacity, we varied both parameters by 20 %, 50 %, and 100 %.

$$IA = 500 * \log(noc) * ahs$$

$$ICC = 500 * noc * ahs$$

Section S4.2 Time step

Because landscapes usually change on a time scale slower than vegetation dynamics (Akcakaya 2001), we changed the habitat suitability map only every five years based on the observation from horticulture that *C. miniata* reaches blooming size in about five years (Pacific Horticulture Society 2020). Shorter time steps (1 or 3 years) required substantially higher computational resources. Sensitivity analysis showed that variation of time step affected the uncertainty range but did not change the directions of trends or the final abundance, see Figure S5.

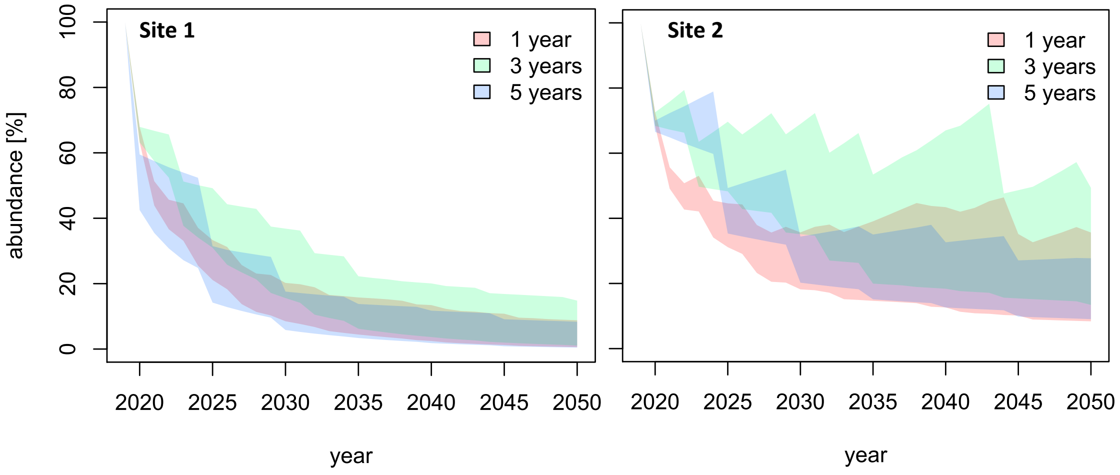

Figure S5: Effect of time step on Clivia miniata metapopulation size in 'CanESM2 RCP2.6, climate change only, no harvest' for site 1 (left) and site 2 (right).

Section S4.3 Neighborhood distance and dispersal

The neighbourhood distance was used to find patches in the habitat suitability (HS) map. Suitable cells (as defined by the HS threshold parameter) that were separated by a distance less than or equal to the neighbourhood distance were regarded to be in the same patch (Akçakaya and Root 2013). The unit of distance assumed was one cell, thus 1 km in our study. For the main paper, we chose 5 km as a compromise between ecological meaningfulness, uncertainty, and computational resources (the number of populations in ND2 is an order of magnitude larger than ND5). Overall, shorter neighbourhood distances (2 or 3 km) had a positive effect on the metapopulation but came with much larger uncertainties, Table S13 and Figure S6. Therefore, our results with ND5 probably underestimate the population size. However, this should not affect the relative effect of different drivers.

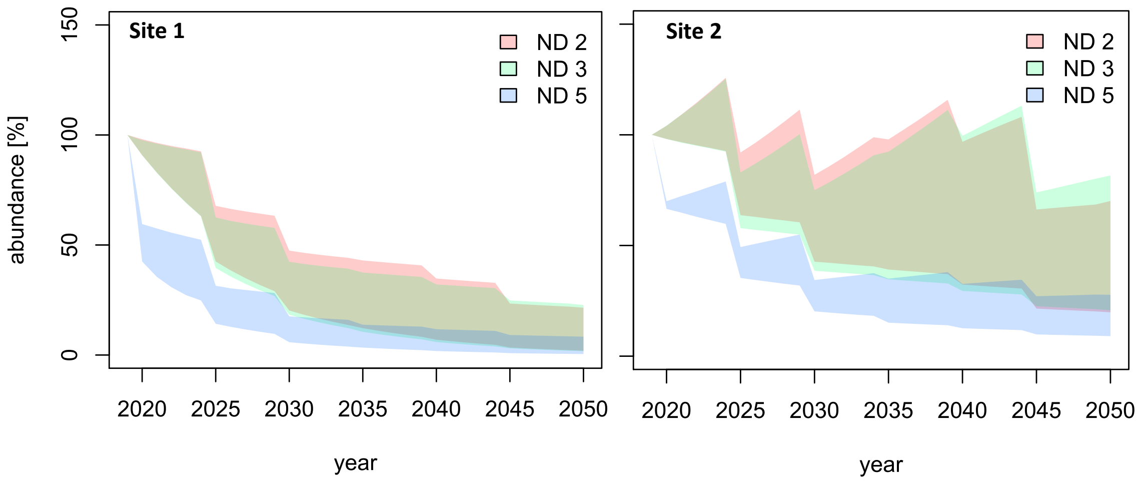

Figure S6: Effect of neighborhood distance on Clivia miniata metapopulation size in 'CanESM2 RCP2.6, climate change only, no harvest' for site 1 (left) and site 2 (right).

Further, we assumed short distance dispersal only, and therefore 5 km allowed us to run simulations without explicit consideration of dispersal. One example of a simulation with dispersal is summarised in Tab. S13. The dispersal function tested with a=0.5 b=5 c=1 D_{max}=50.

$$mij = a\,exp\left(\frac{-D_{ij}^{c}}{b}\right), D \leq D_{max}; \quad 0, D > D_{max}.$$

Section S4.4 Leslie matrix standard deviations

Due to data limitations, we estimated the order of magnitude for year-to-year variability based on previous RAMAS studies on vegetation in South Africa as 20 % for fecundity and 10 % for transitions (Fordham et al. 2012). We tested the sensitivity to increases of 50 % and 100 %. We found that in comparison with the range of uncertainty about the actual Leslie matrix, variations in standard deviations made little difference to the simulations, i.e., variation was dominated by uncertainty.

Table S13: Sensitivity analysis results for C. miniata metapopulation model at the example of 'CanESM2 RCP2.6 CC'. All results are presented in % relative to initial abundance. The 95 percentile is defined as the area between the mean 2.5 percentile -2SD and the mean 97.5 percentile +2SD.

parameter	Variation	2030s		2040s		2050s	
		**Site 1**	**Site 2**	**Site 1**	**Site 2**	**Site 1**	**Site 2**
Reference main paper	-	**12-33**	**24-47**	**3-22**	**16-56**	**1-16**	**12-49**
Initial abundance	*+20%*	12	25-43	5	18-48	2	14-38
	+50%	12-31	23-46	4-21	15-49	1-15	11-39
	+100%	15-31	22-44	4-21	15-43	1-14	11-32
Initial Carrying capacity	*+20%*	12-33	24-48	3-23	16-58	1-16	12-55
	+50%	12-34	25-49	3-23	17-60	1-17	12-62
	+100%	12-35	25-51	3-24	17-61	1-17	12-70
Time step	*1 year*	8-25	18-40	2-18	13-50	1-12	8-43
	3 years	16-44	35-75	4-27	18-76	1-19	13-57
Neighbourhood distance	*2*	20-52	43-86	7-39	32-102	2-26	20-74
	3	18-49	39-80	6-39	30-108	2-29	21-92
dispersal	*on*	12-32	23-47	3-20	15-55	1-14	11-49
St dev Leslie matrix	*+50%*	11-34	23-48	3-23	16-57	1-17	11-15
	+100%	10-36	23-49	3-25	16-57	1-17	11-51

Section S5 Results

Table S14: Loss of C. miniata's suitable habitat area (in %) between the 2020s and 2050s for land cover change only (LC), climate change only (CC), and climate and land cover change combined (CCLC) under RCP2.6 and RCP8.5 for four general circulation models. In the last column, we compared the sum of individual effect sizes (CC+LC) to the simulations with both pressures (CCLC) to test whether the nature of interactions between land cover and climate change was 'synergistic', 'additive', or 'antagonistic'.

**Experiment**	**LC [%]**	**CC [%]**	**CCLC [%}**	**Type of interaction**
**CanESM2-ES RCP2.6**	- 61	- 20	- 75	synergistic
**CanESM2-ES RCP8.5**	- 61	- 13	- 77	antagonistic
**HadGEM2-ES RCP2.6**	- 61	- 7	- 70	antagonistic
**HadGEM2-ES RCP8.5**	- 61	- 16	- 73	synergistic
**MPI-ESM-MR RCP2.6**	- 61	- 7	- 69	antagonistic
**MPI-ESM-MR RCP8.5**	- 61	- 38	- 79	synergistic
**MRI-GGCM3 RCP2.6**	- 61	- 38	- 80	synergistic
**MRI-CGCM3 RCP8.5**	- 61	- 9	- 70	additive

Section S6 Limitations

The accuracy and plausibility of climate scenarios are limited by the quality of the input climate data, the downscaling method, and the omission of extreme events. The main limitation of our land cover scenarios is the uncertainty in future land cover change, which is underpinned by complex interactions of environmental and socioeconomic factors. The predictive power of the SDM and the parameterization of the metapopulation model are also uncertain because of limited data availability on the occurrence, initial abundance, long-term demographics, and species physiological response to changes in the environment. Sensitivity analysis showed that our selection of time step and neighbourhood distance probably underestimates the metapopulation viability. However, this should not affect the relative effect of different drivers. With better estimates of initial abundance and vital rates, we could also better estimate the number of individuals harvested that corresponds to quantities from market surveys (Mander 1998; Mander et al. 2007; Williams et al. 2007). Estimates of demographic parameters could be improved with consideration of multiple species with similar life history if available. Finally, the method does not consider feedbacks between pressures which are crucial for the dynamics of metapopulation (Oliver and Morecroft 2014; Bühne et al. 2021) and the climate-vegetation system (Groner et al. 2018). Because different land cover types have different impacts on surface fluxes of radiation, heat, moisture and momentum, land cover change affects local climate which in turn affects land cover directly though climate and indirectly through people's land use decisions (Duveiller et al. 2020). To fully capture the combined effects of environmental changes and harvesting on biodiversity, the system dynamics need to be captured.

References

Akcakaya, H. Resit. 2001. “Linking Population-Level Risk Assessment with Landscape and Habitat Models.” The Science of the Total Environment 274 (August): 283–91. https://doi.org/10.1016/S0048-9697(01)00750-1.

Akçakaya, H. Reşit, and W.T. Root. 2013. RAMAS GIS: Linking Spatial Data with Population Viability Analysis (Version 6). Setauket, New York: Applied Biomathematics.

Allouche, Omri, Asaf Tsoar, and Ronen Kadmon. 2006. “Assessing the Accuracy of Species Distribution Models: Prevalence, Kappa and the True Skill Statistic (TSS).” Journal of Applied Ecology 43 (6): 1223–32. https://doi.org/10.1111/j.1365-2664.2006.01214.x.

Amatulli, Giuseppe, Sami Domisch, Mao-Ning Tuanmu, Benoit Parmentier, Ajay Ranipeta, Jeremy Malczyk, and Walter Jetz. 2018. “A Suite of Global, Cross-Scale Topographic Variables for Environmental and Biodiversity Modeling.” Scientific Data 5 (March): 180040.

Araújo, Miguel B., and Mark New. 2007. “Ensemble Forecasting of Species Distributions.” Trends in Ecology & Evolution 22 (1): 42–47. https://doi.org/10.1016/j.tree.2006.09.010.

Broxton, Patrick D., Xubin Zeng, Damien Sulla-Menashe, and Peter A. Troch. 2014. “A Global Land Cover Climatology Using MODIS Data.” Journal of Applied Meteorology and Climatology 53 (6): 1593–1605. https://doi.org/10.1175/JAMC-D-13-0270.1.

Bühne, Henrike Schulte to, Joseph A. Tobias, Sarah M. Durant, and Nathalie Pettorelli. 2021. “Improving Predictions of Climate Change–Land Use Change Interactions.” Trends in Ecology & Evolution 36 (1): 29–38. https://doi.org/10.1016/j.tree.2020.08.019.

CHELSA. 2019. http://chelsa-climate.org. Accessed 30 January 2019.

Dixon, Roger. 2011. “Relationships in Clivia.” Clivia 13: 63–81.

Duveiller, Gregory, Luca Caporaso, Raul Abad-Viñas, Lucia Perugini, Giacomo Grassi, Almut Arneth, and Alessandro Cescatti. 2020. “Local Biophysical Effects of Land Use and Land Cover Change: Towards an Assessment Tool for Policy Makers.” Land Use Policy 91 (February): 104382. https://doi.org/10.1016/j.landusepol.2019.104382.

ESGF. 2019. https://esgf-node.llnl.gov/projects/cmip5/. Accessed 19 February 2019

Fick, Stephen E., and Robert J. Hijmans. 2017. “WorldClim 2: New 1-Km Spatial Resolution Climate Surfaces for Global Land Areas.” International Journal of Climatology 37 (12): 4302–15. https://doi.org/10.1002/joc.5086.

Fischer, G., F. Nachtergaele, S. Prieler, H.T. van Velthuizen, L. Verelst, and D. Wiberg. 2008. “Gloal Agro-Ecological Zones Assessment for Agriculture (GAEZ 2008).” IIASA Laxenburg, Austria and FAO, Rome, Italy.

Fordham, Damien A., Tom M. L. Wigley, and Barry W. Brook. 2011. “Multi-model Climate Projections for Biodiversity Risk Assessments.” Ecological Applications 21 (8): 3317–31. https://doi.org/10.1890/11-0314.1.

GBIF.org. 2019. “GBIF Occurrence Download - Clivia Miniata (Lindl.) Verschaff.,” 21 October 2019. https://doi.org/10.15468/dl.1p0bil.

Groner, V. P., T. Raddatz, C. H. Reick, and M. Claussen. 2018. “Plant Functional Diversity Affects Climate–Vegetation Interaction.” Biogeosciences 15 (7): 1947–68. https://doi.org/10.5194/bg-15-1947-2018.

Hanley, J.A., and B.J. McNeil. 1982. “The Meaning and Use of the Area under a Receiver Operating Characteristic (ROC) Curve” 143: 29–36.

Hijmans, R.J., S. Phillips, J. Leathwick, and J. Elith. 2011. “Package ‘Dismo’. Species Distribution Modelling Iwith R.” http://cran.r-project.org/web/packages/dismo/index.html.

IPCC. 2014. “Climate Change 2014: Synthesis Report. Contribution of Working Groups I, II and III to the Fifth Assessment Report of the Intergovernmental Panel on Climate Change [Core Writing Team, R. K. Pachauri and L. A. Meyer (Eds.)].” Geneva, Switzerland: IPCC.

Jentsch, Anke, Jürgen Kreyling, and Carl Beierkuhnlein. 2007. “A New Generation of Climate-Change Experiments: Events, Not Trends.” Frontiers in Ecology and the Environment 5 (7): 365–74. https://doi.org/10.1890/1540-9295(2007)5[365:ANGOCE]2.0.CO;2.

Johnson, Jerald B., and Kristian S. Omland. 2004. “Model Selection in Ecology and Evolution.” Trends in Ecology & Evolution 19 (2): 101–8. https://doi.org/10.1016/j.tree.2003.10.013.

Karger, Dirk Nikolaus, Olaf Conrad, Jürgen Böhner, Tobias Kawohl, Holger Kreft, Rodrigo Wilber Soria-Auza, Niklaus E. Zimmermann, H. Peter Linder, and Michael Kessler. 2017a. “Climatologies at High Resolution for the Earth’s Land Surface Areas.” Scientific Data 4 (September): 170122.

Karger, Dirk Nikolaus, Olaf Conrad, Jürgen Böhner, Tobias Kawohl, Kreft, Rodrigo Wilber Soria-Auza, Niklaus E. Zimmermann, H. Peter Linder, and Michael Kessler. 2017b. “Climatologies at High Resolution for the Earth’s Land Surface Areas.” Dryad Digital Repository. https://doi.org/10.5061/dryad.kd1d4.

Lobo, Jorge M., Alberto Jiménez-Valverde, and Raimundo Real. 2008. “AUC: A Misleading Measure of the Performance of Predictive Distribution Models.” Global Ecology and Biogeography 17 (2): 145–51. https://doi.org/10.1111/j.1466-8238.2007.00358.x.

Mander, Myles. 1998. Marketing of Indigenous Medicinal Plants in South Africa: A Case Study in Kwazulu-Natal. https://doi.org/10.13140/2.1.1073.4084.

Mander, Myles, Lungile Ntuli, Nicci Diederichs, and Khulile Mavundla. 2007. “Economics of the Traditional Medicine Trade in South Africa : Health Care Delivery.” South African Health Review, 2007 (1): 189–96.

McSweeney, C. F., R. G. Jones, R. W. Lee, and D. P. Rowell. 2015. “Selecting CMIP5 GCMs for Downscaling over Multiple Regions.” Climate Dynamics 44 (11): 3237–60. https://doi.org/10.1007/s00382-014-2418-8.

Oliver, Tom H., and Mike D. Morecroft. 2014. “Interactions between Climate Change and Land Use Change on Biodiversity: Attribution Problems, Risks, and Opportunities.” WIREs Climate Change 5 (3): 317–35. https://doi.org/10.1002/wcc.271.

Pacific Horticulture Society. 2020. “Www.Pacifichorticulture.Org.” Https://Www.Pacifichorticulture.Org/Articles/Cultivating-Clivia/. Accessed 11 June 2020.

Phillips, Steven J., Miroslav Dudík, Jane Elith, Catherine H. Graham, Anthony Lehmann, John Leathwick, and Simon Ferrier. 2009. “Sample Selection Bias and Presence-Only Distribution Models: Implications

for Background and Pseudo-Absence Data.” Ecological Applications 19 (1): 181–97. https://doi.org/10.1890/07-2153.1.

Pierce, David W., Tim Barnett, Benjamin Santer, and Peter J Gleckler. 2009. “Selecting Global Climate Models for Regional Climate Change Studies.” Proceedings of the National Academy of Sciences of the United States of America 106 (May): 8441–46. https://doi.org/10.1073/pnas.0900094106.

RHS. 2021. Https://Www.Rhs.Org.Uk/Plants/4036/Clivia-Miniata/Details. Accessed 19 May 2021.

Schulzweida, U. 2019. “CDO User Guide (Version 1.9.6). Doi:10.5281/Zenodo.2558193.”

Seo, Changwan, Thorne James H, Hanna Lee, and Wilfried Thuiller. 2009. “Scale Effects in Species Distribution Models: Implications for Conservation Planning under Climate Change.” Biology Letters 5 (1): 39–43. https://doi.org/10.1098/rsbl.2008.0476.

Stanton, Jessica C., Richard G. Pearson, Ned Horning, Peter Ersts, and H. Resit Akcakaya. 2012. “Combining Static and Dynamic Variables in Species Distribution Models under Climate Change” 3: 349–57. https://doi.org/10.1111/j.2041-210X.2011.00157.x.

Swanevelder, Zacharias Hendrik. 2005. “Diversity and Population Structure of Clivia Miniata Lindl. (Amaryllidacea) : Evidence from Molecular Genetics and Ecology.” University of Pretoria.

Taylor, Karl E., Ronald J. Stouffer, and Gerald A. Meehl. 2011. “An Overview of CMIP5 and the Experiment Design.” Bulletin of the American Meteorological Society 93 (4): 485–98. https://doi.org/10.1175/BAMS-D-11-00094.1.

Vuuren, Detlef P. van, Jae Edmonds, Mikiko Kainuma, Keywan Riahi, Allison Thomson, Kathy Hibbard, George C. Hurtt, et al. 2011. “The Representative Concentration Pathways: An Overview.” Climatic Change 109 (1): 5. https://doi.org/10.1007/s10584-011-0148-z.

Williams, Vivienne L., Ed T.F. Witkowski, and Kevin Balkwill. 2007. “Volume and Financial Value of Species Traded in the Medicinal Plant Markets of Gauteng, South Africa.” International Journal of Sustainable Development & World Ecology 14 (6): 584–603. https://doi.org/10.1080/13504500709469757.

Williams, V.L., J.E. Victor, and N.R. Crouch. 2013. “Red Listed Medicinal Plants of South Africa: Status, Trends, and Assessment Challenges.” South African Journal of Botany 86 (May): 23–35. https://doi.org/10.1016/j.sajb.2013.01.006.

Worldclim. 2019. http://worldclim.org. Accessed 11 February 2019.
